# Elevated Dickkopf 3 Promotes Abdominal Aortic Aneurysm Formation via Modulated Phenotype Switch of Vascular Smooth Muscle Cells

**DOI:** 10.34133/research.0873

**Published:** 2025-09-11

**Authors:** Xuejie Cao, Jinmeng Jia, Qiuyue Gao, Jiaping Tao, Ming Wei, Yanting Song, Hong Wu, Shiyu Jiao, Xinxin Zhu, Xuegong Zhang, Yi Fu, Yuan Wang, Jie Du, Qingbo Xu, Aijuan Qu, Baoqi Yu

**Affiliations:** ^1^ Department of Physiology and Pathophysiology, School of Basic Medical Sciences, Capital Medical University, Key Laboratory of Remodeling-Related Cardiovascular Diseases, Ministry of Education, Laboratory for Clinical Medicine, Capital Medical University, Beijing 100069, China.; ^2^ Bioinformatics Division of BRNIST and Department of Automation, MOE Key Lab of Bioinformatics, Tsinghua University, Beijing 100084, China.; ^3^ Department of Pathology, Beijing Anzhen Hospital Affiliated to Capital Medical University, Beijing 100029, China.; ^4^ Department of Cardiology, The First Affiliated Hospital, Zhejiang University School of Medicine, Hangzhou 310016, China.; ^5^ School of Life Sciences, Center for Synthetic and Systems Biology, Tsinghua University, Beijing 100084, China.; ^6^ Department of Physiology and Pathophysiology, School of Basic Medical Sciences, Peking University, Beijing 100191, China.; ^7^ Beijing Anzhen Hospital of Capital Medical University and Beijing Institute of Heart Lung and Blood Vessel Diseases, Beijing 100029, China.

## Abstract

Abdominal aortic aneurysm (AAA) is a potentially fatal vascular disease with no effective therapeutic intervention. Vascular smooth muscle cell (VSMC) phenotypic switching and elevated matrix metalloproteinase (MMP) levels are key pathogeneses of AAA, although the underlying regulatory mechanisms remain to be fully elucidated. In our study, single-cell RNA-sequencing data analysis demonstrated a substantial elevation in modulated VSMCs in patients with aortic aneurysm, accompanied by Dickkopf 3 (DKK3) up-regulation. Both systemic DKK3 knockout and VSMC-specific DKK3 knockdown led to a marked decrease in both the incidence and mortality of AAA in mice. Reintroduction of DKK3 in *Dkk3*^−/−^*Apoe*^−/−^ mice via adeno-associated virus (AAV) exacerbated AAA development. DKK3 deficiency maintained the contractile phenotype of VSMC and inhibited MMP production. Given the critical role of TGF-β signaling in VSMC phenotypic switching and the progression of AAA, its regulatory mechanisms exhibit spatiotemporal heterogeneity, and the precise underlying mechanisms require further investigation. Next, we aim to investigate the regulators of this pathway. Mechanistically, DKK3 deficiency activates the TGFβ3–Smad2/3 signaling pathway by down-regulating ATF6, thereby inhibiting VSMC phenotype switching. In summary, these findings indicate that DKK3 drives the phenotypic transition of VSMCs to a synthetic phenotype through the ATF6–TGFβ3–Smad2/3 signaling pathway during the development of AAA, which represents a potential target for therapeutic intervention to maintain VSMC homeostasis in AAA.

## Introduction

The global incidence of abdominal aortic aneurysm (AAA) is on the rise [1]. Typically, AAA is asymptomatic, but if it ruptures, the mortality of patients can reach 65% to 85% [2]. While surgical therapies for AAA continue to improve, the absence of effective pharmacotherapy impedes AAA prevention, as the complete pathogenesis remains unclear [3,4]. The pathogenesis of AAA involves a decreased count of normal vascular smooth muscle cells (VSMCs), matrix metalloproteinases (MMPs) degrading extracellular matrix (ECM), accumulation of immune cells, and increased oxidative stress in the vascular wall [2]. Among these phenotypes, VSMC switching is the most critical for aortic aneurysm (AA) formation [5]. During the progression of AA, VSMCs transition from contractile phenotype to synthetic phenotype, and increased levels of cytokines and MMPs are expressed in synthetic VSMCs, which exacerbate disease progression [6,7].

To date, some studies have identified various signaling pathways that are implicated in regulating VSMC phenotypic switching in the AA progression [8]. Notably, in some genetic aortic diseases such as Marfan syndrome (characterized by FBN1 mutation) and Loeys–Dietz syndrome (characterized by TGFBR1/2 mutations), excessive activation of the TGF-β signaling pathway has been observed [9,10]. This aberrant activation leads to increased collagen I level in VSMCs, resulting in vascular stiffness and promoting AA development [11]. On the other hand, in nongenetic AAA, activated TGF-β signaling plays an opposing role: It promotes the transcription of VSMC contraction phenotype marker genes, thereby preventing the progression of AA [12,13]. This striking discrepancy reveals the paradoxical dual roles of the TGF-β pathway in the progression of AAs.

What is the molecular mechanism underlying the divergent function of TGF-β signaling pathway in nongenetic AAA? The complex role of TGF-β in AA formation highlights the necessity of identifying upstream regulators or cofactors that determine pathway specificity, potentially providing novel therapeutic insights for heterogeneous aortic diseases.

​There are 4 known members​ of the Dickkopf (DKK) family, designated as DKK1 to DKK4 [14,15]. Moreover, it has been reported that DKKs can affect cell migration and invasion in cancer by regulating the TGF-β signaling pathway [16,17]. The effects of DKK family members on cardiovascular diseases have been reported in the literature. For instance, DKK1 was found to promote the formation and instability of atherosclerotic plaque by inducing endothelial cell (EC) inflammation and apoptosis [18,19]. DKK2 was demonstrated to enhance neovascularization in the hind limb ischemia and myocardial infarction mouse models [20]. DKK3 has gained recognition​ as a biomarker for cardiovascular disease in recent years [21]. ​Differing from other DKK family components, DKK3 has a distinct structure, with an N-terminal domain before the Cys-1 region and a C-terminal domain after the Cys-2 domain [14]. DKK3 has been implicated in pathological angiogenesis [22–25]. It drives embryonic stem cells, vascular Sca1^+^ progenitor cells, and fibroblasts toward SMC differentiation [26,27]. Moreover, DKK3 ablation in mice was found to prevent atherosclerosis development by alleviation of inflammatory response and the reduction of the level of MMPs [28]. It has been reported that DKK3 stimulated​ SMC differentiation in progenitor cells via the TGF-β signaling pathway, and DKK3 silencing activated the TGF-β signaling pathway [7,27]. However, the impact of DKK3 on AAA formation and whether DKK3 can affect VSMC phenotype switching remain unclear.

The present study examined the hypothesis that DKK3 contributes to the development of AAA. Our results demonstrated that DKK3 level was elevated in the VSMCs of ascending thoracic aortic aneurysm (ATAA) patients compared to other family members. DKK3 promoted VSMC phenotypic switching, up-regulated MMP expression, and accelerated AAA formation and development through the ATF6–TGFβ3–Smad2/3 axis. These results revealed that DKK3 ​represents a promising target for AAA prevention and clinical intervention.

## Results

### DKK3 is considerably up-regulated in human AA patients

**Fig. 1.**
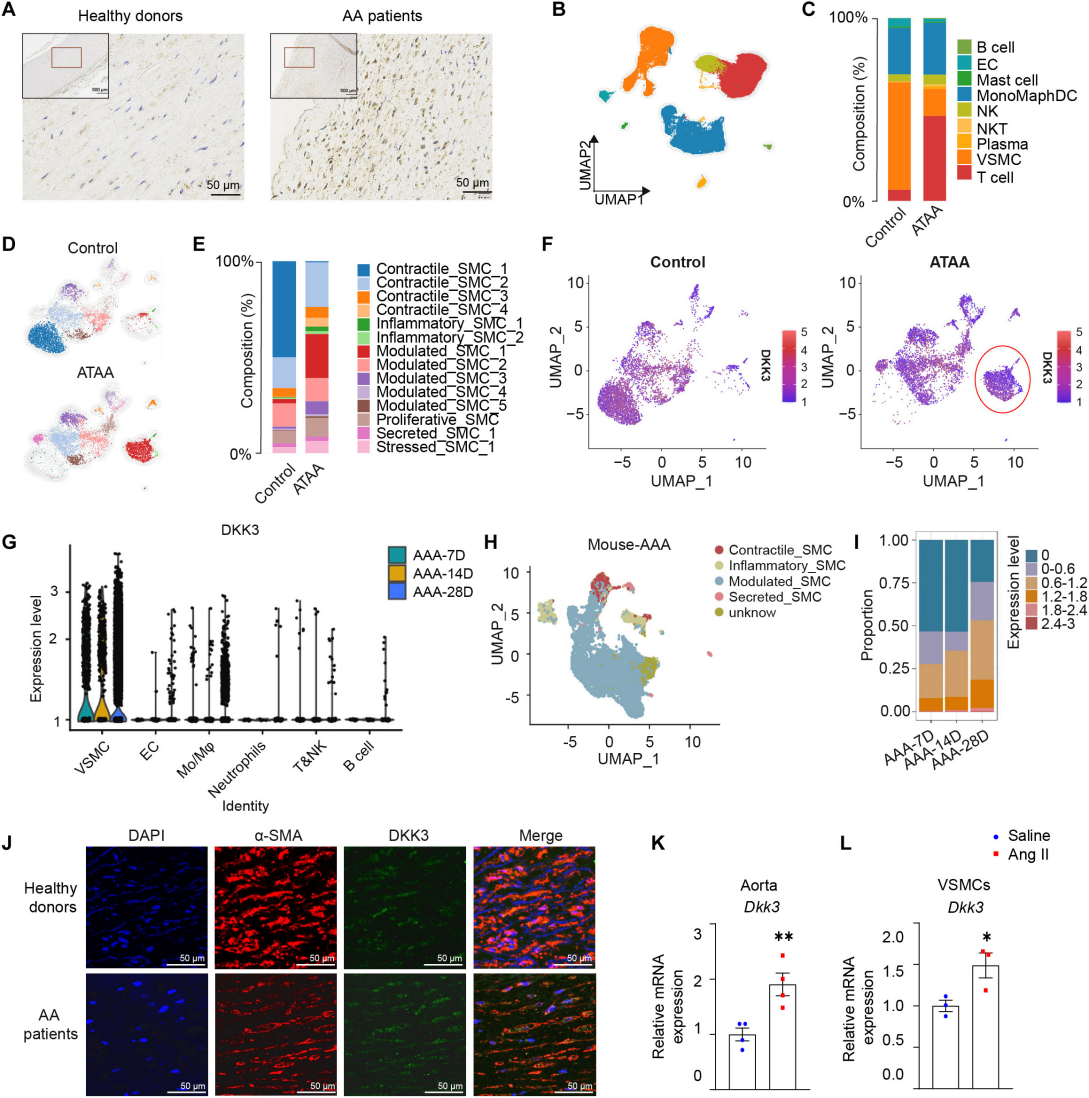
The DKK3 expression was high in AA patients. (A) Immunohistochemical staining to detect DKK3 expression in human normal (*n* = 4) and AA aorta tissues (*n* = 4). Identification of cell clusters and the key factors in AA patients by scRNA-seq analysis of the public dataset GSE155468. (B) UMAP plot showing all cells colored according to all the major cell types. (C) The stacked bar plot represents the proportion of cells in aorta ATAA patients (*n* = 8) and healthy controls (*n* = 3). (D) UMAP plot of VSMC subpopulations of ATAA patients and healthy controls. (E) The stacked bar plot represents the proportion of VSMCs in the aorta of ATAA patients and healthy controls. (F) Feature plots displaying the single-cell gene expression of DKK3 across cell clusters. (G) Violin plot of the differential expression of Dkk3 in different kinds of cells in the aorta of AAA mouse model for 7D (*n* = 5), 14D (*n* = 5), and 28D (*n* = 12). (H) UMAP plots of the SMC subpopulations from the AAA mouse model. (I) The stacked bar plot represents the proportion of cells in the AAA mouse model for 7D, 14D, and 28D. (J) Immunofluorescence stainings of DAPI (blue), α-SMA (red), and DKK3 (green) in human normal aorta (*n* = 6) and AA tissues (*n* = 6). DKK3 mRNA levels in (K) aortas (*n* = 4) and (L) VSMCs (*n* = 3) of *Apoe*^−/−^ mice treated with Ang II or saline. *P < 0.05, **P < 0.01, unpaired Student’s *t* test.

To investigate the level of changes in DKK3 during the development of AAA, we designed a case–control study of 100 AAA patients and 100 age- and sex-matched healthy volunteers, and listed the baseline clinical characteristics of the study (Table S1). The enzyme-linked immunosorbent assay (ELISA) result showed that the plasma DKK3 level of AAA patients (266 ± 317 ng/ml) was significantly higher than that in healthy volunteers (168 ± 141 ng/ml) (Fig. S1A). The multivariate logistic regression analyses were performed, and the result showed that increasing DKK3 level showed a high odds ratio [OR = 1.003, 95% confidence interval (CI) = 1.001 to 1.005, *P* = 0.008]. After the adjustment for gender, age, hypertension, and smoking, increasing DKK3 level still showed an increasing trend in the odds ratio (OR = 1.003, 95% CI = 1.000 to 1.006, *P* = 0.085) (Table S2). Then, we analyzed the microarrays performed with the Illumina HumanHT-12v4 Expression BeadChip platform published in the Gene Expression Omnibus (GEO) database (GSE57691) [29], which contained the results of the abdominal aorta of AAA patients and healthy controls. ​Increased DKK3 expression was observed in the aortas of patients and in both small AAA (aorta diameter ≤55 mm) and large AAA (aorta diameter >55 mm) groups (Fig. S1B to D). Following immunohistochemical staining, we observed that the DKK3-positive area was increased in AA patients than in healthy donors. Moreover, DKK3 was mainly distributed in the media of the aorta (Fig. 1A). Then, single-cell RNA-sequencing (scRNA-seq) analysis was applied to a public dataset GSE155468 [30] containing aortic samples from both ATAA patients and healthy donors. A standard pipeline in Seurat was used for data analysis [30], after which we gained 45,998 cells. The SingleR and CellTypist annotations were utilized in this study. According to the SingleR annotation results, VSMCs and their derived cells, such as stem cell-like cells, osteoblasts, and fibroblasts, are clustered together (Fig. S2A) and exhibit a high degree of similarity (Fig. S2B). CellTypist offers multiple well-trained models for cell annotation (Fig. S2C to E). Based on the combined annotation results from CellTypist and SingleR analyses, we identified cluster2, cluster5, and cluster7 in the Uniform Manifold Approximation and Projection (UMAP) graph as VSMCs with distinct phenotypes (Fig. S2F). Through unsupervised clustering, the scRNA-seq profiles were partitioned into 9 broad lineages of immune and smooth muscle compartments. The lineages were annotated based on cell markers manually curated from the literature and CellMarker 2.0 (Fig. S3A) [30,31]. The 9 broad lineages of compartments included natural killer (NK) cells, natural killer T (NKT) cells, plasma cells, B cells, ECs, mast cells, monocytes, macrophages, dendritic cells (DCs), VSMCs, and T cells (Fig. 1B). We then compared the cellular composition between healthy and ATAA patient samples. As expected, in ATAA patient samples, the proportion of VSMCs was substantially reduced (Fig. 1C)*.* We extracted, reclustered, and annotated VSMCs based on previous public cell markers (Fig. S3B) [32–34]. As a result, we identified a group of VSMCs (Modulated SMC1), the number of which considerably increased in aortic samples of ATAA patients (Fig. 1D and E). Furthermore, we compared the expression of DKK3 among all VSMCs between healthy donors and patients and found that DKK3 was substantially increased in Modulated SMC1 (Fig. 1F).

To further study the changes in DKK3 during the AAA process, we mapped the public scRNA-seq dataset GSE224587 and onto the aforementioned ATAA dataset (GSE155468) to identify the VSMC clusters of abdominal aortas in AAA patients (Fig. S3C). ​Results demonstrated substantially elevated DKK3 expression within VSMCs of the AAA center compared with the proximal AAA, especially in Modulated_SMC (Fig. S3D and E). Furthermore, single-cell analysis of public datasets GSE152583 [35] and GSE221789 [33] (the aortas of the AAA mouse model) confirmed that a substantially higher DKK3 expression was found in VSMCs than in ECs, macrophages, and other inflammatory cells, and the expression was increased along with the AAA progression (Fig. 1G). Then, we first adopted a reference-based integration approach to identify counterparts of mouse VSMCs in humans by mapping human data onto mouse clusters (Fig. 1H). The stacked bar plot represents the proportion of modulated SMCs with different DKK3 expression and their changes (7D, 14D, 28D). The results showed that DKK3 was considerably increased over AAA progression (Fig. 1I). We also observed that the proportion of DKK3 in ECs was slightly increased during AAA development in the mouse model. However, in the human aorta, the expression of DKK3 in ECs of ATAA patients was even lower than that of healthy controls (Fig. S3F and G). In general, DKK3 was mainly expressed in VSMCs and up-regulated along with the AA progression. Then, we further observed the levels of DKK1 and DKK2 in various cell types of human aorta through scRNA-seq analysis, and observed the low expression of DKK1 and DKK2 in different types of cells (Fig. S3H to J).

Next, immunofluorescence staining was performed to further identify the location of DKK3, and the results showed that DKK3 is mainly colocalized with VSMCs and can also colocalized with EC and macrophages in vascular wall. The expression is increased in VSMCs of AA patients (Fig. 1J and Fig. S4A and B). We also found that the level of DKK3 is significantly enhanced in both the aorta and VSMCs of *Apoe*^−/−^ mice treated with angiotensin II (Ang II) (Fig. 1K and L).

These results demonstrated that DKK3 is increased during AAA and is likely implicated in the pathogenesis of AAA.

### DKK3 deficiency attenuates the development of Ang II-induced AAA

To elucidate the function of DKK3 in the AAA formation, we constructed Ang II-induced AAA models on *Dkk3*^−/−^*Apoe*^−/−^ mice and their littermate *Dkk3*^+/+^*Apoe*^−/−^ mice as controls. Firstly, quantitative polymerase chain reaction (qPCR) and Western blot were performed to detect *Dkk3* mRNA (Fig. S5A) and DKK3 protein levels separately in the aorta and heart (Fig. S5B and C). ELISA was performed to measure plasma DKK3 levels as well (Fig. S5D). These results demonstrated that the *Dkk3* gene was disrupted in the *Dkk3*^−/−^*Apoe*^−/−^ mice.

**Fig. 2.**
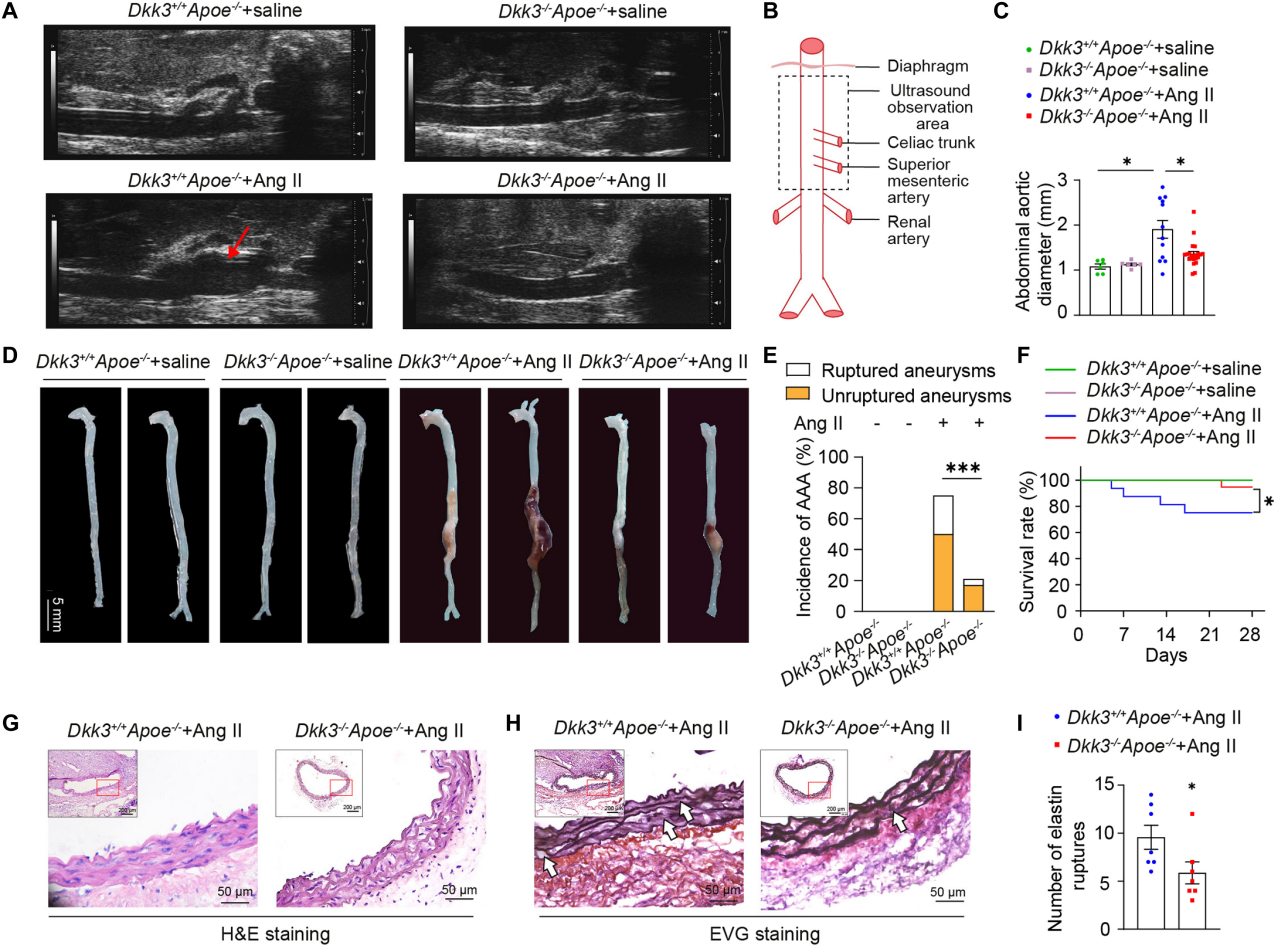
Deficiency of DKK3 alleviated AAA development. (A) B-mode ultrasound of abdominal aorta in *Dkk3*^+/+^*Apoe*^−/−^ and *Dkk3*^−/−^*Apoe*^−/−^ mice treated with Ang II or saline. Arrows indicate the dilated aorta. (B) Schematic diagram of ultrasound location in suprarenal segment of the abdominal aorta. (C) Statistical analysis of maximal abdominal aortic diameters (*Dkk3*^+/+^*Apoe*^−/−^ + saline, *n* = 6; *Dkk3*^−/−^*Apoe*^−/−^ + saline, *n* = 6; *Dkk3*^+/+^*Apoe*^−/−^ + Ang II, *n* = 12; *Dkk3*^−/−^*Apoe*^−/−^ + Ang II, *n* = 22). *P < 0.05, two-way ANOVA. (D) Representative photos of the aortas from *Dkk3*^−/−^*Apoe*^−/−^ and *Dkk3^+/+^Apoe*^−/−^ mice treated with Ang II. (E) Incidence rate of AAA in each group (*Dkk3*^+/+^*Apoe*^−/−^ + saline, *n* = 6; *Dkk3*^−/−^*Apoe*^−/−^ + saline, *n* = 6; *Dkk3*^+/+^*Apoe*^−/−^ + Ang II, *n* = 16; *Dkk3*^−/−^*Apoe*^−/−^ + Ang II, *n* = 23). ***P < 0.001, χ^2^ test. (F) Survival rate of mice in each group (*Dkk3*^+/+^*Apoe*^−/−^ + saline, *n* = 6; *Dkk3*^−/−^*Apoe*^−/−^ + saline, *n* = 6; *Dkk3*^+/+^*Apoe*^−/−^ + Ang II, *n* = 16; *Dkk3*^−/−^*Apoe*^−/−^ + Ang II, *n* = 23). *P < 0.05, Gehan–Breslow–Wilcoxon test. Representative pictures of (G) H&E staining and (H) EVG staining with the abdominal aortas (*n* = 7 of each group). Scale bars, 200 μm and 50 μm. Arrows indicate elastin degradation areas. (I) Quantification of the number of elastin breaks per vessel (*n* = 7 of each group). *P < 0.05, unpaired Student’s *t* test.

Ang II was intravenously administered to *Dkk3*^−/−^*Apoe*^−/−^ and *Dkk3*^+/+^*Apoe*^−/−^ mice at 1,000 ng/(kg × min) for 4 weeks to establish the AAA model. Then ultrasonography of the suprarenal segment of the abdominal aorta was performed for the measurement of the maximal diameter of the abdominal aorta (Fig. 2A and B). The results indicated that the maximum abdominal aortic diameter was larger in *Dkk3*^+/+^*Apoe*^−/−^ mice compared with *Dkk3*^−/−^*Apoe*^−/−^ mice treated with Ang II (Fig. 2C). During Ang II infusion, 75% of *Dkk3*^+/+^*Apoe*^−/−^ mice exhibited AAA and 25% had aortic rupture, whereas 22% of *Dkk3*^−/−^*Apoe*^−/−^ mice experienced AAA and only 4% developed aortic rupture. There was no AA in the mice of the 2 groups treated with saline (Fig. 2D and E). Deficiency of DKK3 significantly extends the survival time of mice (Fig. 2F). Hematoxylin and eosin (H&E) and elastic van Gieson (EVG) staining showed that DKK3 deficiency ameliorated Ang II-induced elastin fragmentation (Fig. 2G to I). These results indicate that DKK3 deficiency remarkably prevented AAA ​progression in Ang II-induced *Apoe*^−/−^ mice.

To further confirm the role of DKK3 in the development of AAA, *Dkk3*^−/−^*Apoe*^−/−^ mice were injected with adeno-associated virus (AAV)-DKK3 (pHBAAV2/9-CMV-m-DKK3-3xflag-T2A-mcherry) for 1.5 × 10^12^ vector genomes (vg)/ml in 100-μl total volume. The expression of DKK3 was measured 4 weeks after transfection by qPCR and immunofluorescence staining (Fig. S6A and B). We then treated *Dkk3*^−/−^*Apoe*^−/−^ mice injected with AAV-DKK3 with Ang II. After AAV-DKK3 injection, the maximal abdominal aortic diameter in the *Dkk3*^−/−^*Apoe*^−/−^ mice had an increased tendency (Fig. S6C and D) and the incidence of AAA was 57%, which is significantly enhanced compared with the control group (10%) (Fig. S6F and G). But neither group of mice died from AAA rupture (Fig. S6E). H&E and EVG staining showed that treatment with AAV-DKK3 elevated Ang II-induced elastin fragmentation (Fig. S6H to J). These results showed that systematic DKK3 expression could potentiate the development of AAA induced by Ang II.

### Non-bone marrow-derived DKK3 deletion exhibits more marked effects on the inhibition of AAA development

In order to further verify the source of DKK3 that affects the development of AAA, bone marrow transplantation was performed. Irradiated (8.5 Gy) *Dkk3*^+/+^*Apoe*^−/−^ and *Dkk3*^−/−^*Apoe*^−/−^ mice received bone marrow cells from *Dkk3^+/+^Apoe*^−/−^ and *Dkk3*^−/−^*Apoe*^−/−^ mice. After 4 weeks, these chimeric mice were treated with Ang II 1,000 ng/(kg × min) (Fig. S7A). ​Following 4-week Ang II administration, the maximum diameter of abdominal aorta in the group of *Dkk3*^+/+^*Apoe*^−/−^ mice transplanted with *Dkk3*^−/−^*Apoe*^−/−^ mouse bone marrow (BMT ^DKO→*Apoe*−/−^) was larger than that in *Dkk3*^−/−^*Apoe*^−/−^ mice transplanted with *Dkk3*^+/+^*Apoe*^−/−^ mouse bone marrow (BMT ^*Apoe*−/−→DKO^) (Fig. S7B and C). Furthermore, 40% of BMT ^*Apoe*−/−→DKO^ mice developed AAA, and 20% had AAA rupture, whereas in the group of BMT ^DKO→*Apoe*−/−^, 80% mice had AAA and 20% died from AAA rupture during Ang II infusion. However, no intergroup disparity in survival time was observed in these 2 groups.​ As a positive control, the incidence rate (83%) and mortality rate (33%) of AAA in *Dkk3*^+/+^*Apoe*^−/−^ mice transplanted with *Dkk3*^+/+^*Apoe*^−/−^ bone marrow (BMT ^*Apoe*−/−→*Apoe*−/−^) were much higher than those in the negative control group of *Dkk3*^−/−^*Apoe*^−/−^ mice transplanted with *Dkk3*^−/−^*Apoe*^−/−^ bone marrow (BMT ^DKO→DKO^), which exhibited the lowest incidence rate (33%) without rupture (Fig. S7D to F). On the aortic cross sections, H&E and EVG staining showed fewer elastin ruptures in the artic wall of BMT ^*Apoe*−/−→DKO^ mice, compared with the BMT ^DKO→*Apoe*−/−^ mice (Fig. S7G to I). These results suggested that DKK3 deficiency in non-bone marrow cells, such as vascular cells, rather than bone marrow-derived cells, contributed more substantially to the development of AAA.

### VSMC-specific DKK3 knockdown inhibits AAA development

**Fig. 3.**
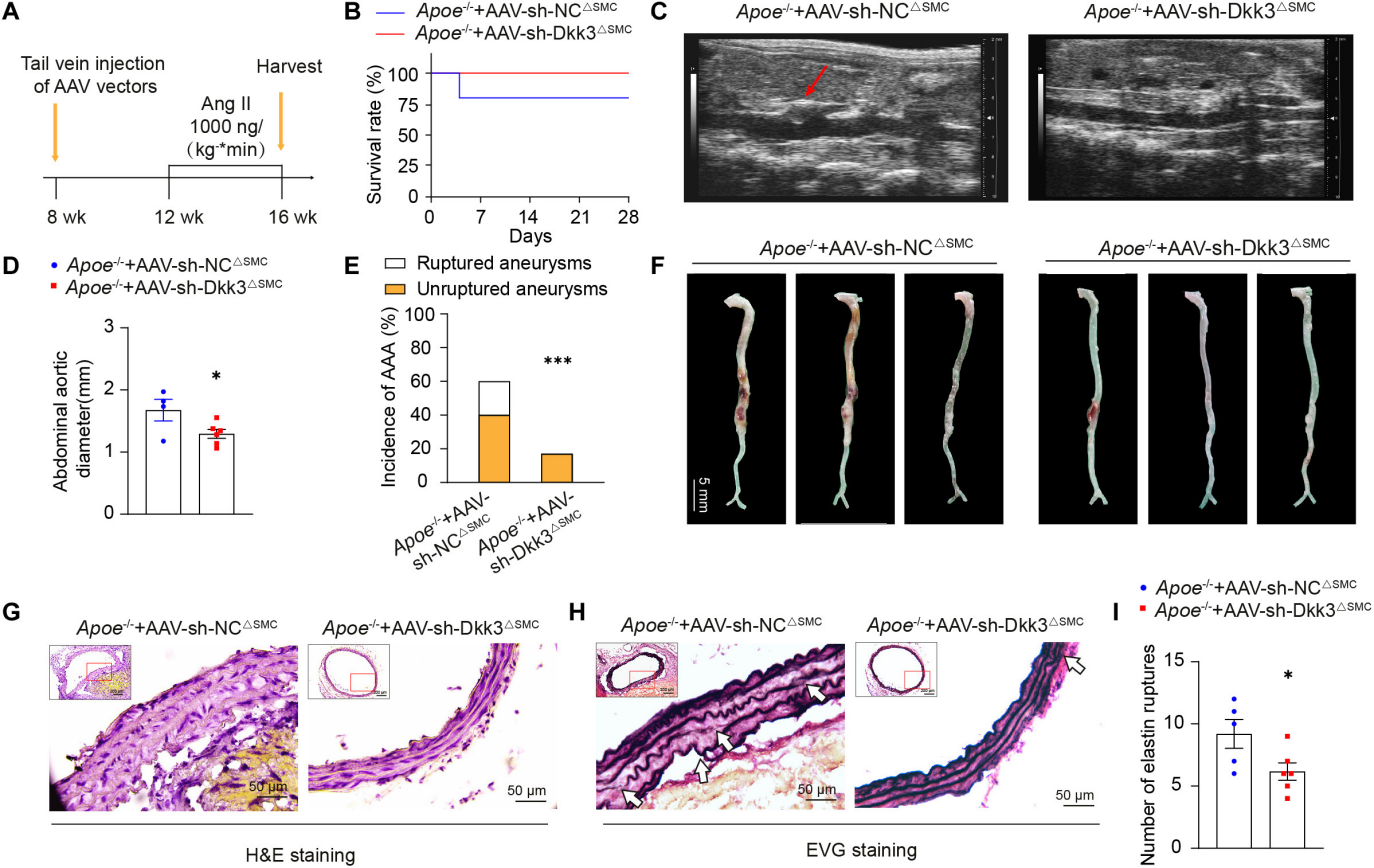
VSMC-specific DKK3 knockdown inhibited AAA development. (A) Schematic diagram of constructing AAA mouse model with VSMC-specific DKK3 knockdown. (B) Survival rate of mice in each group (*Apoe*^−/−^ + AAV-sh-NC^∆SMC^ + Ang II, *n* = 5; *Apoe*^−/−^ + AAV-sh-Dkk3^∆SMC^ + Ang II, *n* = 6), Gehan–Breslow–Wilcoxon test. (C) B-mode ultrasound of abdominal aorta in the *Apoe*^−/−^ + AAV-sh-NC^∆SMC^ mice and *Apoe*^−/−^ + AAV-sh-Dkk3^∆SMC^ mice treated with Ang II. Arrows indicate dilated aorta. (D) Statistical analysis of maximal abdominal aortic diameters in each group (*Apoe*^−/−^ + AAV-sh-NC^∆SMC^ + Ang II, *n* = 4; *Apoe*^−/−^ + AAV-sh-Dkk3^∆SMC^ + Ang II, *n* = 6). *P < 0.05, unpaired Student’s *t* test. (E) Incidence rate of AAA in each group (*Apoe*^−/−^ + AAV-sh-NC^∆SMC^ + Ang II, *n* = 5; *Apoe*^−/−^ + AAV-sh-Dkk3^∆SMC^ + Ang II, *n* = 6). ***P < 0.001, χ^2^ test. (F) Representative photos of the aortas from *Apoe*^−/−^ + AAV-sh-NC^∆SMC^ mice and *Apoe*^−/−^ + AAV9-sh-Dkk3^∆SMC^ mice treated with Ang II. The arrow indicates aneurysm area. Representative pictures of (G) H&E staining and (H) EVG staining with the abdominal aortas (*Apoe*^−/−^ + AAV-sh-NC^∆SMC^ + Ang II, *n* = 5; *Apoe*^−/−^ + AAV-sh-Dkk3^∆SMC^ + Ang II, *n* = 6). Scale bars, 200 μm and 50 μm. Arrows indicate elastin degradation areas. (I) Quantification of the number of elastin breaks per vessel section (*Apoe*^−/−^ + AAV-sh-NC^∆SMC^ + Ang II, *n* = 5; *Apoe*^−/−^ + AAV-sh-Dkk3^∆SMC^ + Ang II, *n* = 6). *P < 0.05, unpaired Student’s *t* test.

Our previous results showed that non-bone marrow-derived DKK3 played a more important role in AAA development, and DKK3 is mainly colocalized with VSMCs (Fig. 1J). To further validate the role of DKK3 in VSMCs during AAA development, we utilized AAV2/9 vectors carrying short hairpin RNA (shRNA)–DKK3 with an SM22α promoter (pHBAAV2/9-SM22a-mir30-m-DKK3) to specifically knock down DKK3 in VSMCs in Ang II-induced *Dkk3*^+/+^*Apoe*^−/−^ mice. Confirmation of DKK3 knockdown at both gene and protein levels was achieved 4 weeks post-transfection (Fig. S8A and B). Then, these mice received continuous Ang II administration of 1,000 ng/(kg × min) for 4 weeks (Fig. 3A). We found that VSMC-specific knockdown of DKK3 prolonged the survival time (Fig. 3B) and significantly reduced the maximum diameter of the abdominal aorta (Fig. 3C and D). It resulted in a reduced incidence rate (17%) of AAA compared to the control group, which exhibited rates of 60% and 20%, respectively (Fig. 3E and F). H&E and EVG staining indicated that VSMC-specific DKK3 knockdown mitigated Ang II-induced elastin fragmentation (Fig. 3G to I).

### DKK3 deficiency inhibits Ang II-induced modulated VSMC phenotype switching and MMP expression

**Fig. 4.**
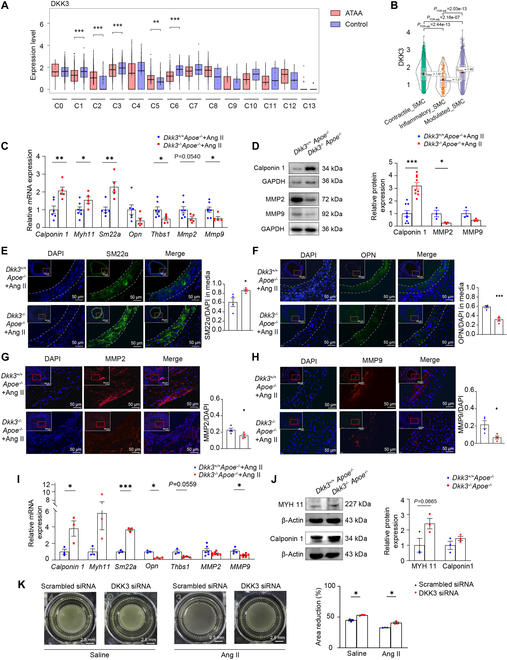
DKK3 deficiency inhibited VSMC phenotypic switching and MMP expression*.* (A) Boxplot of the differential expression of DKK3 of VSMCs in the aorta of ATAA patients and healthy controls (C0: Contractile_SMC_1, C1: Contractile_SMC_2, C2: Modulated_SMC_1, C3: Modulated_SMC_2, C4: Proliferative_SMC, C5: Stressed_SMC_1, C6: Contractile_SMC_3, C7: Modulated_SMC_3, C8: Contractile_SMC_4, C9: Secreted_SMC_1, C10: Inflammatory_SMC_1, C11: Inflammatory_SMC_2, C12: Modulated_SMC_4, C13: Modulated_SMC_5). **P < 0.01, ***P < 0.001, unpaired Student’s *t* test. (B) Violin plot of the differential expression of DKK3 of contractile (*n* = 4,722 cells), inflammatory (*n* = 174 cells), and modulated (*n* = 2,503 cells) types of VSMC in the aorta of ATAA patients. (C) VSMC contractile markers and synthetic markers in the aorta in *Dkk3*^+/+^*Apoe*^−/−^ and *Dkk3*^−/−^*Apoe*^−/−^ mice treated with Ang II examined by qPCR (*n* = 5 to 7). *P < 0.05, **P < 0.01, unpaired Student’s *t* test. (D) The protein level of Calponin, MMP2, and MMP9 in the aorta in *Dkk3*^+/+^*Apoe*^−/−^ and *Dkk3*^−/−^*Apoe*^−/−^ mice treated with Ang II (*n* = 3 to 9). *P < 0.05, ***P < 0.001, unpaired Student’s *t* test. Immunofluorescence staining of (E) DAPI (blue) and SM22α (green), (F) DAPI (blue) and OPN (green), (G) DAPI (blue) and MMP2 (red), and (H) DAPI (blue) and MMP9 (red). *P < 0.05, ***P < 0.001, unpaired Student’s *t* test. (I) mRNA levels of VSMC contractile and synthetic markers in *Dkk3*^+/+^*Apoe*^−/−^ or *Dkk3*^−/−^*Apoe*^−/−^ VSMCs examined by qPCR (*n* = 3 to 7). *P < 0.05, ***P < 0.001, unpaired Student’s *t* test. (J) Protein levels of MYH11 and Calponin 1 in *Dkk3*^+/+^*Apoe*^−/−^ or *Dkk3*^−/−^*Apoe*^−/−^ VSMCs (*n* = 3). Unpaired Student’s *t* test. (K) Representative images and statistical results of the gel contraction assay for HASMCs transfected with scrambled siRNA or DKK3 siRNA, and quantification of the area reduction was shown in percentages (*n* = 3). *P < 0.05, unpaired Student’s *t* test. Scale bars, 2.5 mm.

VSMC phenotypic switching and ECM degradation are critical factors in AAA development. Our previous results also showed that the contractile VSMC was reduced and modulated VSMC was increased in the aorta of ATAA patients (Fig. 1E), and the expression of DKK3 was substantially up-regulated in VSMCs of AA patients (Fig. 1J). Therefore, we compared the expression of DKK3 among all VSMC clusters between healthy controls and patients and found that DKK3 was significantly increased in Modulated SMC1 (cluster-C2) (Fig. 4A), while the levels of DKK1 and DKK2 were not significantly different between the 2 groups (Fig. S9A and B). Moreover, we found that in the aorta of ATAA patients, DKK3 levels in both Contractile_SMC and Modulated_SMC were higher than those in Inflammatory_SMC, with the highest expression level observed in Modulated_SMC (Fig. 4B).

Then, we further studied whether DKK3 affects VSMC phenotype during the development of AAA by using in vivo experiments. qPCR performed on the aortas of Ang II-infused mice showed that the contractile markers such as *Calponin 1*, *Myh11*, and *Sm22a* mRNAs were higher, while the synthetic markers like *Opn*, *Thbs1*, as well as *Mmp2* and *Mmp9* mRNAs were lower in *Dkk3*^−/−^*Apoe*^−/−^ mice compared with *Dkk3*^+/+^*Apoe*^−/−^ mice (Fig. 4C). The results of Western blot also indicated that the protein level of the contractile marker Calponin 1 was elevated in the aorta of *Dkk3*^−/−^*Apoe*^−/−^ mice, and the level of MMP2 and MMP9 was decreased (Fig. 4D). Immunofluorescence staining showed that the contractile VSMC marker SM22α was increased, while the synthetic VSMC marker OPN and the MMP2 and MMP9 levels were decreased in the aortic wall of *Dkk3*^−/−^*Apoe*^−/−^ mice compared with *Dkk3*^+/+^*Apoe*^−/−^ mice infused with Ang II (Fig. 4E to H).

In vitro, VSMCs isolated from the aortas of *Dkk3*^−/−^*Apoe*^−/−^ or *Dkk3*^+/+^*Apoe*^−/−^ mice were stimulated with Ang II separately. The expression of contractile markers was significantly up-regulated, while the synthetic markers were down-regulated in the *Dkk3*^−/−^*Apoe*^−/−^ VSMCs. Furthermore, the levels of *Mmp2* and *Mmp9* mRNAs in the *Dkk3*^−/−^*Apoe*^−/−^ VSMCs were also less than those in *Dkk3^+/+^Apoe*^−/−^ VSMCs (Fig. 4I). Western blot analysis revealed that the protein levels of contractile markers such as MYH11 and Calponin 1 had an increasing trend, while the synthetic markers such as fibronectin and THBS1 had a decreasing tendency in the VSMCs of *Dkk3*^−/−^*Apoe*^−/−^ mice (Fig. 4J and Fig. S9C). A cell contraction assay also revealed increased contractility in human aortic smooth muscle cells (HASMCs) transfected with DKK3 small interfering RNA (siRNA) (Fig. 4K).

Taken together, DKK3 can promote phenotype switching of VSMC toward a synthetic state and increase the MMP expression both in vivo and in vitro*.*

### Lack of DKK3 activates TGF-β signaling pathway during the progression of AAA

**Fig. 5.**
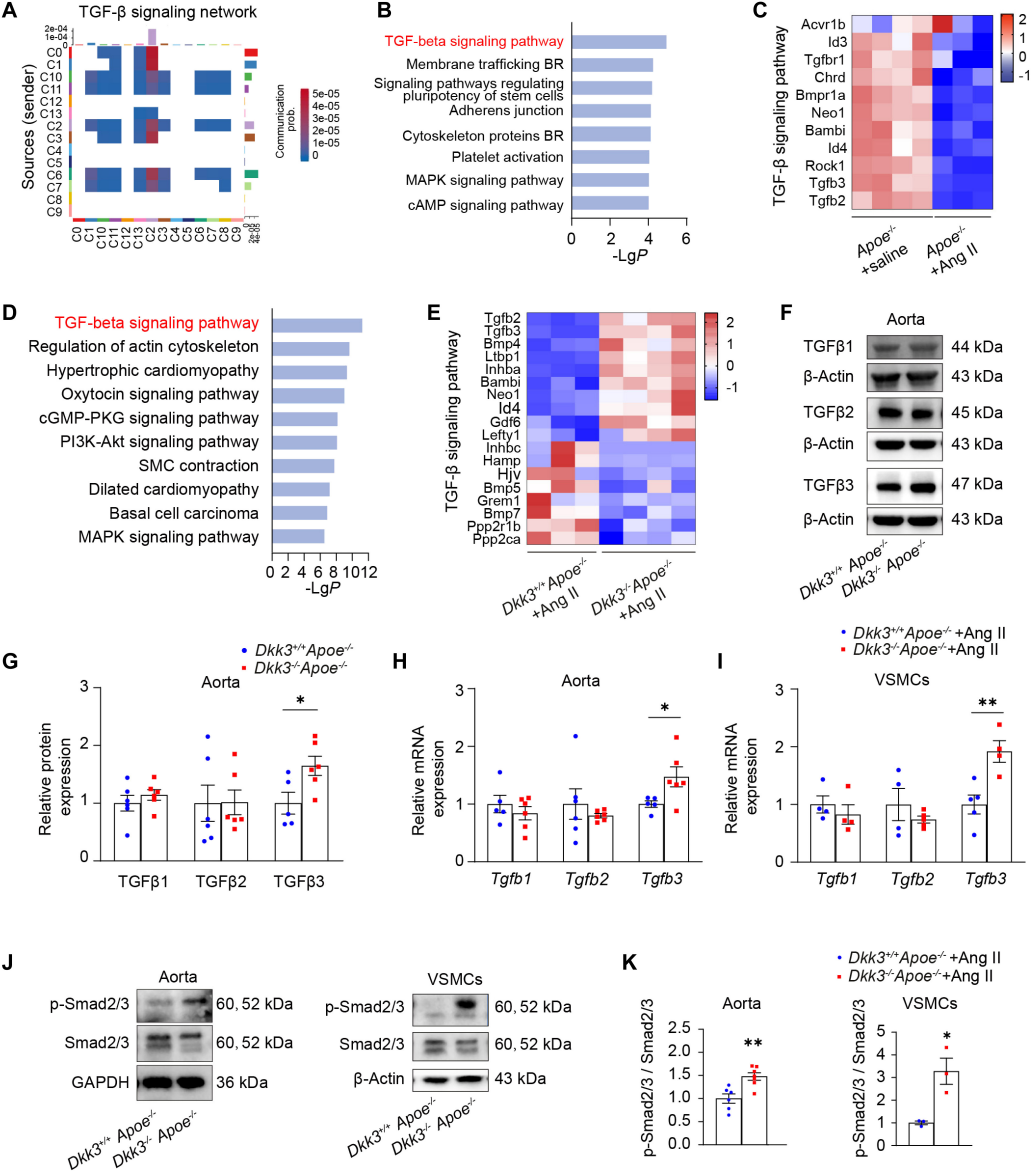
DKK3 deficiency inhibited VSMC phenotypic switching through the TGF-β signaling pathway. (A) Identification of cell clusters in AA patients by scRNA-seq analysis of the public dataset GSE155468. Cell–cell communication analysis among VSMC subtypes. (B) Enrichment analysis of DEGs compared the aortas of *Apoe*^−/−^ mice treated with Ang II and saline using KEGG. (C) Heatmap of differentially regulated genes related to the TGF-β signaling pathway *Apoe*^−/−^ mice treated with Ang II and saline. (D) Enrichment analysis of DEGs compared the aortas of *Dkk3*^+/+^*Apoe*^−/−^ and *Dkk3*^−/−^*Apoe*^−/−^ mice treated with Ang II using KEGG. (E) Heatmap of differentially regulated genes related to the TGF-β signaling pathway. (F) Protein levels of TGFβ1, TGFβ2, and TGFβ3 in aortas of *Dkk3*^+/+^*Apoe*^−/−^ and *Dkk3*^−/−^*Apoe*^−/−^ mice. (G) Quantification of Western blot analysis for TGFβ1, TGFβ2, and TGFβ3 normalized to β-actin (*n* = 5 to 6). *P < 0.05, unpaired Student’s *t* test. The mRNA levels of *Tgfb1*, *Tgfb2*, and *Tgfb3* in the (H) aorta and (I) VSMCs of *Dkk3*^+/+^*Apoe*^−/−^ and *Dkk3*^−/−^*Apoe*^−/−^ mice treated with Ang II (*n* = 4 to 6). *P < 0.05, **P < 0.01, unpaired Student’s *t* test. (J) p-Smad2/3 level in aortas and VSMCs of *Dkk3*^+/+^*Apoe*^−/−^ and *Dkk3*^−/−^*Apoe*^−/−^ mice treated with Ang II. (K) Quantification of Western blot analysis for p-Smad2/3 normalized to Smad2/3 (*n* = 3 to 6). *P < 0.05, **P < 0.01, unpaired Student’s *t* test.

To further study the mechanism of the VSMC phenotypic switching mediated by DKK3, we further conducted the scRNA-seq analysis in dataset GSE155468. We characterized Modulated SMC1 with its molecular functions and intercellular cell–cell communication networks with other cell groups. We predicted the cell–cell communication network of all VSMCs and found that Modulated SMC1 (cluster-C2) was the major receiving cell of the TGF-β signaling network (Fig. 5A). It has been reported that ​the TGF-β/Smad axis coordinates VSMC phenotypic switching [36]. There are 3 subtypes of TGF-β family ligands, including TGFβ1, TGFβ2, and TGFβ3, and they can bind to their receptors (TGFβRI and TGFβRII), initiating a cascade that activates Smad2/3, which then translocates to the nucleus to modulate downstream genes [37,38]. NicheNet analysis revealed marked differences in the activity of the TGFB3-ACVRL1 signaling pathway in Modulated SMC1 (cluster-C2) (Fig. S10A). We compared the expression of TGFβ1 to TGFβ3 among all VSMCs between healthy controls and patients and found that TGFβ3 was significantly decreased in Modulated SMC1 (cluster-C2) (Fig. S10B and C). The expression of TGFβ1 and TGFβ2 had no significant difference between the Modulated SMC1 (cluster-C2) and other phenotypes of VSMCs of healthy controls and patients (Fig. S10D and G). Furthermore, we treated *Apoe*^−/−^ mice with Ang II and conducted RNA-seq, and the results demonstrated TGF-β signaling pathway down-regulation in the aorta of AAA mice compared with *Apoe*^−/−^ mice treated with saline, with TGFβ2 and TGFβ3 being the main down-regulated genes (Fig. 5B and C).

To delineate the mechanistic basis of DKK3-induced AAA, we conducted RNA-seq analysis on the aortas of *Dkk3*^+/+^*Apoe*^−/−^ mice and *Dkk3*^−/−^*Apoe*^−/−^ mice treated with Ang II. The results revealed the different gene expression profiles between the 2 groups of mice (Fig. S10D), in which 1,747 genes were up-regulated and 2,042 showed reduced expression in the aorta of *Dkk3*^−/−^*Apoe*^−/−^ mice compared with *Dkk3*^+/+^*Apoe*^−/−^ mice (Fig. S10E). Bioinformatics analysis identified these differentially expressed genes (DEGs) that were correlated with the TGF-β signaling pathway, guanosine 3′,5′-monophosphate (cGMP)–protein kinase G (PKG) signaling pathway, and phosphatidylinositol 3-kinase (PI3K)–Akt signaling pathway. Notably, among them, the TGF-β signaling pathway exhibited the most significant enrichment (Fig. 5D). Moreover, compared to the *Dkk3*^+/+^*Apoe*^−/−^ mice, *Tgfb2* and *Tgfb3* were significantly up-regulated instead of *Tgfb1* in the TGF-β signaling pathway in the aorta of *Dkk3*^−/−^*Apoe*^−/−^ mice, as indicated by the results of our RNA-seq analysis (Fig. 5E). Enhanced TGF-β3 protein levels were validated by Western blot in the aorta of *Dkk3*^−/−^*Apoe*^−/−^ mice (Fig. 5F and G). qPCR confirmed the up-regulation of *Tgfb3* in both the aorta and VSMCs of *Dkk3*^−/−^*Apoe*^−/−^ mice (Fig. 5H and I). Then, Western blot analysis was performed and the results showed that the ​significantly elevated phosphorylated Smad2/3 level was quantified in the aorta and VSMCs of *Dkk3*^−/−^*Apoe*^−/−^ mice as compared with the *Dkk3*^+/+^*Apoe*^−/−^ group (Fig. 5J and K).

DKK3 is a member of the DKK family, which are antagonists of the Wnt signaling pathway [15]. Western blot was performed to measure the level of β-catenin (canonical Wnt pathway) and p-JNK (noncanonical Wnt signaling pathway). However, comparable β-catenin and p-JNK levels were maintained in both aorta and VSMCs regardless of the presence or absence of DKK3 (Fig. S10F and G).

These results showed that DKK3 deficiency up-regulated the TGF-β signaling pathway during the progression of AAA.

### DKK3 deficiency maintains VSMC contractile phenotype by up-regulating the TGF-β–Smad3 signaling pathway through ATF6 down-regulation

**Fig. 6.**
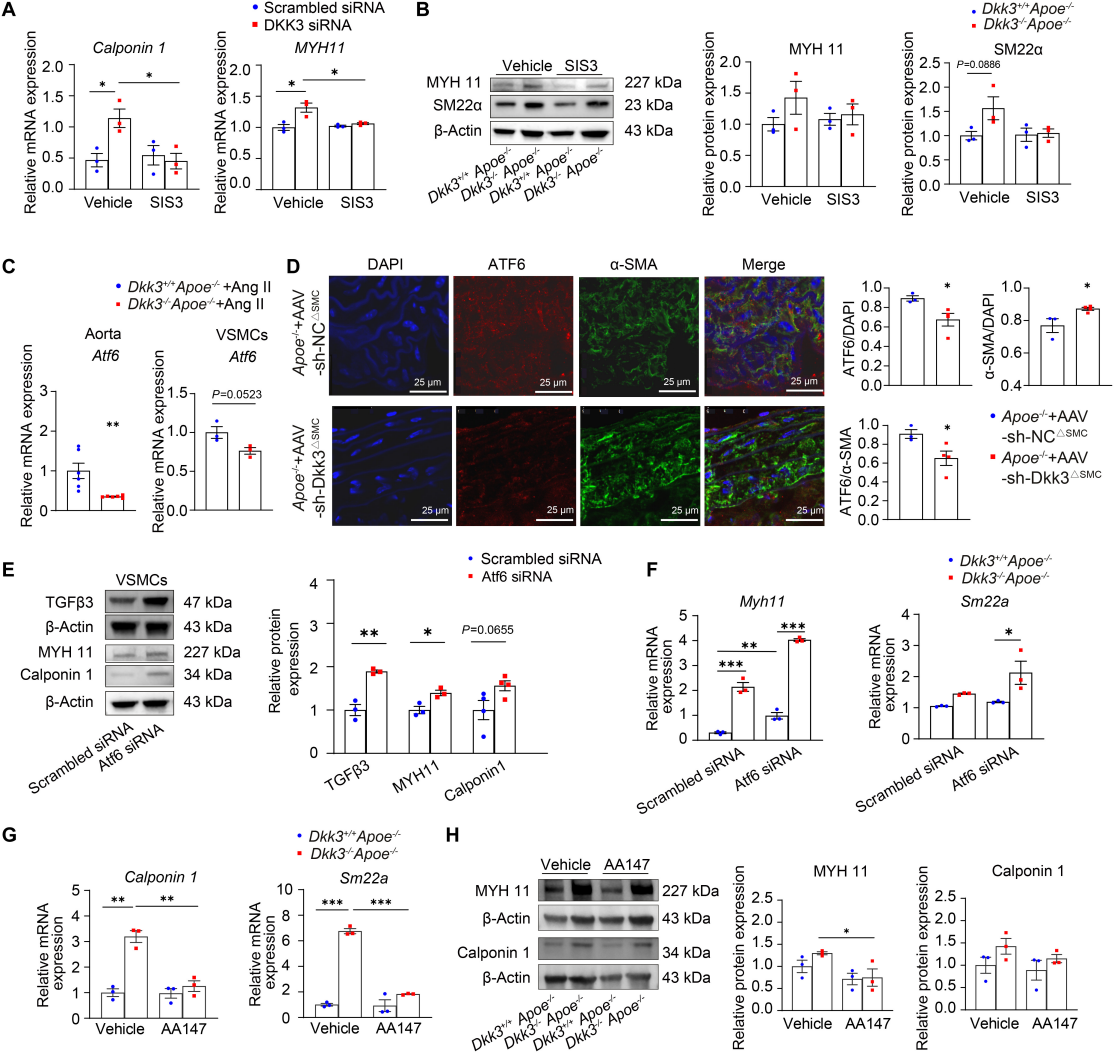
DKK3 regulates the TGF-β–Smad3 signaling pathway through ATF6. (A) mRNA levels of *Calponin 1* and *MYH11* in HASMCs transfected with scrambled siRNA or DKK3 siRNA before treatment with SIS3 (*n* = 3). *P < 0.05, two-way ANOVA. (B) VSMC contractile markers detected by Western blot in *Dkk3*^+/+^*Apoe*^−/−^ and *Dkk3*^−/−^*Apoe*^−/−^ VSMCs treated with SIS3 (*n* = 3). Two-way ANOVA. (C) mRNA levels of *Atf6* in the aorta and VSMCs of *Dkk3*^+/+^*Apoe*^−/−^ and *Dkk3*^−/−^*Apoe*^−/−^ mice (*n* = 3 to 6). **P < 0.01, unpaired Student’s *t* test. (D) Immunofluorescence stainings of DAPI (blue), ATF6 (red), and α-SMA (green) in the aorta of *Apoe*^−/−^ + AAV-sh-NC^∆SMC^ mice and *Apoe*^−/−^ + AAV-sh-Dkk3^∆SMC^ mice treated with Ang II (*n* = 3 to 4). *P < 0.05, unpaired Student’s *t* test. (E) Protein levels of TGFβ3 and VSMC contractile markers in *Apoe*^−/−^ VSMCs transfected with scrambled siRNA or Atf6 siRNA (*n* = 3). *P < 0.05, **P < 0.01, unpaired Student’s *t* test. (F) mRNA levels of *Myh11* and *Sm22a* in *Dkk3*^+/+^*Apoe*^−/−^ and *Dkk3*^−/−^*Apoe*^−/−^ VSMCs transfected with scrambled siRNA or Atf6 siRNA (*n* = 3). *P < 0.05, **P < 0.01, ***P < 0.001, two-way ANOVA. (G) mRNA levels of *Calponin 1* and *Sm22a* in *Dkk3*^+/+^*Apoe*^−/−^ and *Dkk3*^−/−^*Apoe*^−/−^ VSMCs treated with AA147 (*n* = 3). **P < 0.01, ***P < 0.001, two-way ANOVA. (H) VSMC contractile markers detected by Western blot in *Dkk3*^+/+^*Apoe*^−/−^ and *Dkk3*^−/−^*Apoe*^−/−^ VSMC treatment with AA147 (*n* = 3). *P < 0.05, two-way ANOVA.

To verify whether DKK3 regulated VSMC phenotype switching through the TGF-β–Smad signaling pathway, SIS3 (a Smad2/3 signaling pathway inhibitor) was applied. The results revealed that SIS3 offsets the effect of DKK3 deficiency on maintaining the VSMC phenotype, and the mRNA and protein levels of VSMC contractile markers were decreased after the stimulation of SIS3 (Fig. 6A and B).

Next, we aim to further clarify the mechanism by which DKK3 inhibits the TGF-β signaling pathway to regulate VSMC phenotypic switching. We performed high-resolution weighted gene coexpression network analysis (hdWGCNA) on all VSMCs and identified a C2-specific module (yellow module) enriched in cluster-C2 cells (Modulated SMC1) (Fig. S11A). This approach enabled the identification of transcriptional programs specific to distinct SMC subsets, allowing functional characterization of C2. Genes from the yellow module were subsequently input into the STRING database (v11.5) to construct a protein–protein interaction (PPI) network using a combined score threshold of ≥0.4. We then applied the Markov clustering algorithm (MCL) to delineate topological submodules within the PPI network, revealing densely connected functional units. Degree and eigenvector centrality were computed for each node using the R implementation of NetworkX to assess direct connectivity and overall influence within the network. TGFB3, SERPINE1, and THBS1 exhibited high scores in both metrics, highlighting their central roles as regulatory hubs within the fibrotic module (Fig. S11B). Besides, our previous studies confirmed the irreplaceable contributions of DKK3 in the differentiation of VSMCs from stem cells through the activation of transcription factor 6 (ATF6) [26,27,39]. Given the central position of TGFB3 and the established role of ATF6, we incorporated regulatory nodes HSPA5 and ATF6 based on their known roles in endoplasmic reticulum (ER) stress signaling [40,41]. These genes were not initially present in the module but were integrated based on experimentally validated pathways and expression co-occurrence. Similarly, DKK3 was introduced as a WNT pathway modulator connected to LEFTY2 and FHL2 [42]. A directed signaling graph was constructed linking DKK3–ATF6–HSPA5/SERPINE1–TGFB3, combining expression network topology, literature-derived edges, and known biological pathways. This framework supports the hypothesis that DKK3 regulates TGFB3 expression via ATF6-mediated ER stress (Fig. S11C and D).

Moreover, down-regulation of *Atf6* was observed in the aorta and VSMCs of *Dkk3*^−/−^*Apoe*^−/−^ mice (Fig. 6C). Subsequently, through the result of immunofluorescence staining, we observed that VSMC-specific DKK3 knockdown significantly decreased ATF6 expression of VSMCs but increased the level of VSMC contraction phenotype marker α-smooth muscle actin (α-SMA), and DKK3 knockdown reduced the proportion of VSMCs expressing ATF6 (Fig. 6D). To further investigate how DKK3 affects ATF6 expression, we conducted co-immunoprecipitation (Co-IP) in *Apoe*^−/−^ VSMCs with specific antibodies against DKK3 followed by immunoblotting (IB) with antibodies against ATF6, and the result indicated that ATF6 can be efficiently co-immunoprecipitated with DKK3 (Fig. S11E). Previous studies also reported that cytokines can play a role in regulating transcription in the nucleus [43,44]. We then designed the promoter sequences of *ATF6* and conducted chromatin immunoprecipitation (ChIP)–qPCR in HASMC using specific antibodies against DKK3. The results of ChIP-qPCR and conventional PCR (c-PCR) showed that DKK3 was highly enriched on the promoters of *ATF6* (Fig. S11F and G)*.*

Then, *Atf6* siRNA was transfected into the *Apoe*^−/−^ VSMCs and the results substantiated that *Atf6* knockdown up-regulated the level of TGFβ3 and up-regulated levels of VSMC contractile markers (Fig. 6E). qPCR results showed that *Atf6* knockdown could further increase the expression of VSMC contraction markers under DKK3 deficiency (Fig. 6F). To further verify the regulatory effect of ATF6 on VSMC phenotypic switching, VSMCs were treated with ATF6 agonist AA147 10 μM for 48 h. qPCR and Western blot revealed that​ AA147 treatment decreased the level of VSMC contractile markers (Fig. 6G and H).

To further clarify the effect of DKK3–ATF6–TGFβ3 axis on VSMC phenotype transformation within in vivo experiments, we conducted additional in vivo experiments by administering AA147 to *Dkk3*^−/−^*Apoe*^−/−^ mice stimulated with Ang II. Immunofluorescence staining was performed to detect the expression of ATF6 (Fig. S11H). We found that AA147 treatment reversed the elevated levels of TGFβ3 caused by DKK3 deficiency, substantially reduced the level of α-SMA and SM22α, and increased the level of OPN (Fig. S11I to K). The result indicates that DKK3–ATF6–TGFβ3 can also regulate VSMC phenotype transformation in vivo.

These findings suggested that ATF6 might act as a key modulator in the DKK3-activated TGF-β signaling pathway and VSMC phenotype switching. In summary, these results revealed that DKK3 regulates VSMC phenotypic switching mainly through the ATF6–TGFβ3–Smad3 axis.

## Discussion

​Results from the present study demonstrated that the level of DKK3 was elevated in the aorta tissue and plasma of AA patients. Systemic deletion of DKK3 and VSMC-specific DKK3 knockdown in *Apoe*^−/−^ mice prevented AAA formation and rupture. Furthermore, the lack of DKK3 stabilized the VSMC contractile phenotype and reduced MMP production in AAA. Mechanistic studies revealed that DKK3 deficiency inhibited the VSMC phenotypic switching to modulated phenotype through the TGF-β/Smad3 signaling pathway, decreased the production of MMPs, and further alleviated the rupture and degradation of elastin. Taken together, our current study showed that DKK3 presents a promising target​ for AAA prevention and treatment.

As a lethal cardiovascular disease, AAA demonstrates elevated morbidity and mortality [2]. Currently, surgery is the main treatment for AAA. However, no effective method has been demonstrated to prevent or treat the progression of AAs [2,3]. The phenotypic transition of VSMCs is a crucial aspect of AAA pathogenesis [5]. This suggests that regulating the VSMC phenotype switching could potentially serve as a treatment strategy for AAA. In our research, by analyzing the scRNA-seq data from a public database, we observed that Modulated SMC1 demonstrated marked up-regulation in the aortas of ATAA patients, concomitant with elevated levels of DKK3. The DKK3 level was increased in the aorta of AA patients, especially in aortic VSMCs.

Previous studies found that DKK3 can induce embryonic stem cell, vascular Sca1^+^ stem cell, and fibroblast differentiation into SMCs [26,27]. Moreover, the absence of DKK3 decreased the level of MMPs and contributed to the reduction of atherosclerosis [28]. VSMC phenotypic switching and apoptosis, and increased MMP levels are crucial for AAA development. Then, we validated the pivotal contribution of DKK3 in the development of AAA in a mouse model. We found that DKK3 deficiency inhibited AAA progression through preservation of the contractile phenotype in VSMC and by down-regulating the MMP level.

VSMC exhibits a high degree of plasticity in various pathologies [8,45]. In vascular diseases such as atherosclerosis, restenosis, AAA, and intracranial aneurysms, VSMCs undergo phenotypic transition and transition to multiple pathological phenotypes such as synthetic, inflammatory, and osteogenic phenotypes [30,36,46]. This process involves a decrease in contractile marker abundance, including SM22α, MYH11, and Calponin 1, alongside an increase in synthetic marker abundance such as THBS1 and OPN [47–49]. Synthetic VSMCs produce elevated levels of cytokines and MMPs, thereby promoting vascular remodeling [6]. Previous studies showed that arsenic trioxide and M2 macrophage-derived exosomes can regulate the phenotype switching and dedifferentiation, and inhibit the development of vascular remodeling diseases [50,51]. This indicates that targeting the regulation of VSMC phenotype holds promise as a therapeutic strategy. According to our research, DKK3 was highly expressed in contractile, inflammatory, and modulated phenotypes of VSMCs. Among them, DKK3 exhibited the highest expression in the modulated phenotype, which is associated with AAA progression. In our study, DKK3 deficiency decreased the VSMC phenotypic switch both in vivo and in vitro, thereby preventing the development of AAA.

Previous studies have shown that TGF-β signaling plays an important role in the VSMC phenotypic switching. Activated TGF-β signaling promotes the transcription of VSMC contraction phenotype marker genes, thereby preventing the progression of AA [12,13]. However, overactivity of TGF-β signaling pathway was observed in the Marfan syndrome (MFS) mouse model induced by Fbn1 mutation [52]. Administration of TGF-β neutralizing antibodies delayed aortic dilation in 7- to 15-week-old Marfan mice, while using this therapy before aneurysm formation can accelerate disease progression [53,54]. The pleiotropy of the TGF-β signaling pathway potentially associates with its effects on different stages of disease development [55]. Due to the pleiotropy and complexity of the TGF-β signaling pathway in aneurysms, the specific regulation of the TGF-β signaling pathway during AAA development requires further study.

DKK3 represents a canonical DKK family, which is one of the Wnt signaling pathway antagonists. Recent studies have revealed that DKK3 can also affect various vascular cells and participate in multiple vascular diseases through TGF-β/Smad2/3 signaling pathways [23,27,56]. Enrichment analysis using Kyoto Encyclopedia of Genes and Genomes (KEGG) revealed that DKK3 deficiency increased the TGF-β, PI3K-Akt, and mitogen-activated protein kinase (MAPK) signaling pathways in the aorta. Accumulated studies support the involvement of these signaling pathways in regulating VSMC phenotype transition [36,57,58]. Among them, the TGF-β signaling pathway was the most significantly enriched and is known to ameliorate AAA progression [59]. Given our previous findings that DKK3 did not affect the Wnt signaling pathway after Ang II stimulation in vivo and in vitro, we focused on the TGF-β signaling pathway and found that DKK3 deficiency increased Smad2/3 phosphorylation stimulated by Ang II. SIS3 treatment counteracted the effect of DKK3 depletion on maintaining the VSMC contractile phenotype. These findings revealed that DKK3 regulates VSMC phenotypic switching through the TGF-β/Smad3 signaling pathway during AAA pathogenesis, whereas the mechanistic basis for the regulation of DKK3 in TGF-β signaling during VSMC phenotypic switching requires further investigation.

Our prior research has found that DKK3 regulated the TGF-β signaling pathway via ATF6 to induce stem cell differentiation into VSMCs [26,27,60]. Notably, it has also been reported that ATF6 levels were elevated in ER stress-induced transformation toward an osteogenic VSMC phenotype [61]. Building on these findings, we further investigated whether ATF6 mediates DKK3-dependent phenotypic switching of VSMCs during AAA progression. We analyzed the public scRNA-seq dataset GSE155468 using STRING-based network reconstruction and MCL clustering. This analysis identified ATF6 as a central node downstream of DKK3 and forming a DKK3–ATF6–HSPA5/SERPINE1–TGFB3 signaling axis. We further confirmed that DKK3 regulated VSMC phenotypic switching to modulate phenotype during AAA development mediated by ATF6, and observed that the lack of DKK3 decreased the level of ATF6. Mechanistically, the down-regulation of ATF6 led to an increase in TGFβ3, followed by the activated TGF-β–Smad2/3 signaling pathway, which maintained VSMC contractile phenotype. These results demonstrated that DKK3 mediates VSMC phenotypic switching via the ATF6–TGF-β3–Smad2/3 axis.

To elucidate how DKK3 regulates ATF6, we performed Co-IP and ChIP-qPCR. The results revealed that DKK3 may regulate ATF6 through 2 distinct mechanisms: (a) DKK3 physically interacts with the ATF6 protein (Fig. S11E). As demonstrated in previous studies, secretory proteins can also bind to proteins in the cytoplasm and exert their effects. For example, prostate apoptosis response-4 (par-4) can bind to atypical protein kinase C (aPKC) in the cytoplasm to inhibit nuclear factor κB (NF-κB) activation and induce apoptosis [62]. However, the precise binding sites involved in the DKK3–ATF6 interaction remain to be fully elucidated. (b) DKK3 may directly bind to the ATF6 promoter, significantly regulating its transcription (Fig. S11F), as evidenced by the reduced ATF6 mRNA level upon DKK3 knockdown (Fig. 6C). These findings align with reports that certain secreted proteins (cytokines and growth factors) can translocate to the nucleus to regulate gene expression. For instance, fibroblast growth factor-1 (FGF-1) enters cells and localizes to the nucleus to reduce the expression of p21 in breast cancer tissues [43,44]. However, the subcellular site of DKK3–ATF6 protein interaction (cytoplasm or nucleus) remains unclear due to ATF6 dynamic processing during ER stress: full-length ATF6 (FL-ATF6) translocation to the Golgi apparatus for proteolytic cleavage, generating the active N-terminal fragment (nATF6), which translocates to the nucleus to regulate target gene expression before degradation by the 26*S* proteasome [63–65]. The potential functional implications of DKK3–ATF6 interaction depend on its subcellular context: (a) Binding to FL-ATF6 in the cytoplasm, DKK3 may modulate ATF6 processing and activation efficiency. (b) Binding to nATF6 in the nucleus, DKK3 could potentially influence its stability. Our present work may suggest a dual regulatory mode in which DKK3 modulates ATF6: DKK3 promotes transcription of ATF6 gene and directly binds to ATF6 protein. The relative contribution of each mechanism under different pathophysiological contexts warrants further investigation.

Human epidemiological and animal studies have established hypertension as a key risk factor for AAA pathogenesis [66,67]. A previous study by Busceti et al. [68] reported that DKK3 regulates blood pressure (BP) in an endothelium-dependent manner via the vascular endothelial growth factor (VEGF)/Akt/endothelial nitric oxide synthase (eNOS) axis. Here, we demonstrate a novel protective role of DKK3 deficiency in AAA development. Firstly, DKK3 deficiency does not universally elevate blood pressure: Busceti’s study observed blood pressure elevation only in a specific, non-drug-induced disease model, while both their Ang II low-dose study (0.3 to 0.7 mg/kg/day for 7 d) in *Dkk3*^−/−^ mice and our robust model (1.4 mg/kg/day for 28 d) in *Dkk3*^−/−^*Apoe*^−/−^ mice showed comparable BP to that of their littermate control. Furthermore, without Ang II treatment, *Dkk3*^−/−^*Apoe*^−/−^ mice exhibited no significant BP difference from their *Apoe*^−/−^ littermate controls, unlike the difference reported between *Dkk3*^−/−^ mice and their C57BL/6 wild-type littermate control. This discrepancy likely reflects the distinct genetic backgrounds: the C57BL/6 wild-type mice represent a relatively normal state in which DKK3 deficiency alone may cause baseline hypertension, whereas *Apoe*^−/−^ mice inherently exhibit marked dyslipidemia and spontaneous endothelial dysfunction/susceptibility to atherosclerosis, creating a pro-hypertensive milieu that may mask or alter the specific contribution of Dkk3 deficiency to baseline BP regulation [69,70]. Collectively, these findings suggest that DKK3 deficiency contributes to hypertension only under certain conditions. In recent years, nonhypertensive pathways have been demonstrated in elastase-induced and CaPO_4_-induced AAA mouse models, such as homocysteine–AT1 receptor-mediated AAA development and SAMD4A-KDM2B-regulated VSMC phenotype transition in AAA progression [71,72]. Thus, our finding that DKK3 deficiency protects against AAA via VSMC phenotype modulation is not contradictory to high blood pressure (HBP)-associated AAA pathogenesis, uncovering a previously unrecognized DKK3 role.

In our study, we also found the DKK3 expression in macrophages and ECs. However, our results of scRNA-seq analysis showed that the expression of DKK3 in VSMCs was substantially higher than that in ECs, macrophages, and other inflammatory cells (Fig. 1G). The results of immunofluorescence staining also showed that DKK3 is mainly colocalized with VSMCs compared with ECs and macrophages (Fig. 1J and Fig. S4A and B). Moreover, previous studies have shown that although EC dysfunction and inflammatory response are the pathological mechanisms of AAA, the loss of VSMC and phenotypic transformation are the most important pathogeneses of AAA. So, in our study, we pay more attention to the effect of DKK3 on VSMC. Further studies are needed to explore the effect of DKK3 on ECs and macrophages in the progression of AAA.

Regarding the limitations of our study, several aspects warrant careful consideration. Firstly, we demonstrated that DKK3 level was enhanced in both the peripheral blood and the aorta of AA patients. The possible reason for the elevation of plasma DKK3 level may be caused by an increase in the DKK3 level of VSMC. DKK3 is a secreted glycoprotein, and an increased DKK3 level in VSMCs leads to enhanced secretion of DKK3 into tissue fluid and blood. However, the underlying mechanism regulating DKK3 expression throughout the pathogenesis of AAA remains unclear, necessitating further studies. In addition, more animal experiments and preclinical studies are necessary to translate our research results into clinical applications and make DKK3 a therapeutic candidate for AAA in clinical practice.

**Fig. 7.**
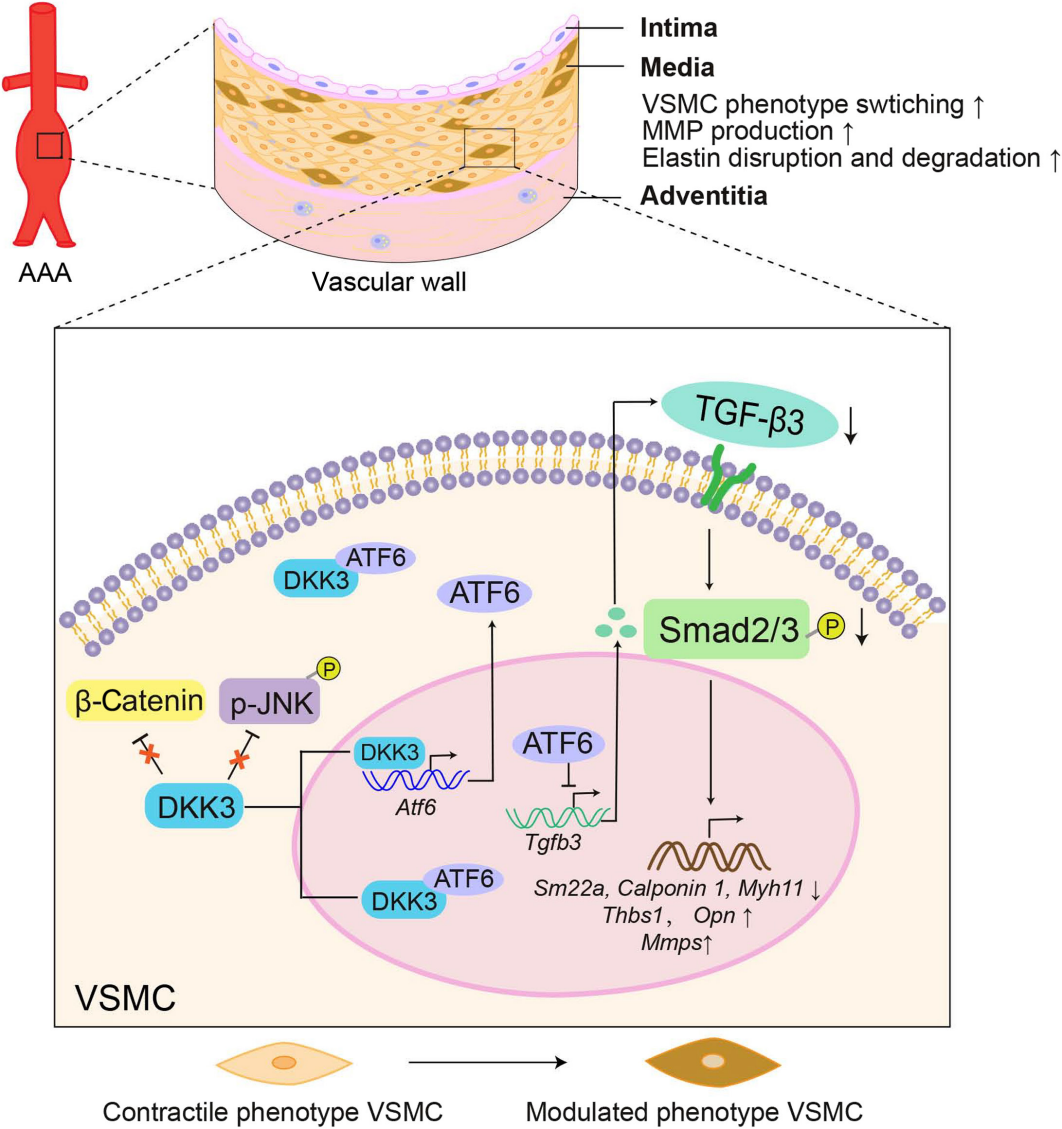
Schematic image of DKK3-mediated regulation of VSMC phenotypic switching during AAA development. DKK3 up-regulates ATF6 expression via 2 mechanisms: transcriptional activation by binding to the ATF6 promoter and posttranslational stabilization through direct protein interaction, potentially occurring in the cytoplasm (with full-length ATF6) or the nucleus (with cleaved nATF6). The accumulated ATF6 represses TGFβ3 transcription, thereby inhibiting the TGF-β/Smad2/3 signaling pathway. This cascade promotes VSMC phenotype switching and increases MMP levels, ultimately exacerbating AAA progression.

The results of our current study elucidated the role of DKK3 in AAA. DKK3 deficiency prevented Ang II-induced AAA development by reducing MMP levels and inhibiting VSMC phenotypic switching to modulated phenotype via the ATF6–TGF-β3–Smad2/3 axis (Fig. 7). Inhibition or blocking of DKK3 could also be a promising clinical treatment for AAA.

## Methods

### Human aortic samples and ethics

Human aortic samples used for paraffin sectioning and staining were collected following patient consent under approved institutional protocol by the ethics committee of Beijing Anzhen Hospital, Capital Medical University (Z2023SY007), and the research complied with the Declaration of Helsinki and the International Conference on Harmonization Guidelines for Good Clinical Practice, with written informed consent acquired from all participants. AA tissue specimens obtained in surgery were collected from AA patients, and all patients provided written informed consent. Normal aortic tissue specimens were obtained from organ donors.

The human plasma used for ELISA detection comes from a case–control study prior to this study. Adult patients with AAA were recruited from Beijing Anzhen Hospital. AAA diagnosis was established via the discharge diagnosis in patients’ medical records and using imaging data. Patients meeting any criterion below were excluded:​ (a) patients in pregnancy; (b) received erythrocyte concentrates, whole blood, or platelets within 10 d prior to blood sample collection; (c) aortic trauma or pseudoaneurysm; and (d) with active cancer, heart failure history, renal impairment, severe respiratory disorders, or other end-stage diseases. Healthy control subjects were recruited from communities of Beijing Anzhen hospital, without major systemic disorders (ischemic heart disease, malignancy, respiratory disorders, active infections). Age- and sex-matched healthy individuals were collected. Blood samples of AAA were collected before surgery. Blood samples underwent immediate centrifugation for plasma isolation, with cryopreservation at −80 °C pending analysis. This research received approval from the Beijing Anzhen Hospital Ethics Review Board, with written informed consent obtained from all enrolled patients​ (approval number: 2014006X). The investigation adhered to the Declaration of Helsinki ethical principles.

### Mouse AAA model and ethics

*Apoe*^−/−^ mice were procured from Beijing Vital River Laboratory Animal Technology Company (Beijing, China). *Dkk3*^−/−^ mice were generated as described previously [73]. *Apoe*^−/−^ mice were crossed with *Dkk3*^−/−^ mice, and heterozygous offspring were mated to obtain *Dkk3^+/+^Apoe*^−/−^ and *Dkk3*^−/−^*Apoe*^−/−^ mice. Mouse genotypes were determined via PCR analysis of tail snip specimens, with primer details listed (Table S3). The mice were maintained at 22 °C, and water and irradiated feed were provided without restriction.

Twelve-week-old male *Dkk3*^+/+^*Apoe*^−/−^ and *Dkk3*^−/−^*Apoe*^−/−^ mice were used for Ang II-induced AAA model. The mice were anesthetized with 2.5% isoflurane. Ang II (A9525, Sigma-Aldrich, St. Louis, MO, USA)-loaded micro-osmotic pumps (ALZET DURECT 1004, Durect Corp., Cupertino, CA, USA) were surgically implanted subcutaneously at a delivery rate of 1,000 ng*kg^−1^min^−1^ for 28 d. The mice were euthanized by CO_2_ inhalation, and the aortas were harvested after 28 d.

The abdominal aortas were surgically resected and immersion-fixed in 4% paraformaldehyde. Residual aortic segments underwent snap-freezing in liquid nitrogen prior to storage at −80 °C until further processing. Animal handling and experimental protocols adhered to the Institute of Laboratory Animal Resources guidelines and the regulations approved by the Capital Medical University Animal Care and Use Committee (AEEI-2020-158). The experiments complied with the Guide for the Care and Use of Laboratory Animals published by the US National Institutes of Health (NIH Publication No. 85-23, revised 1996).

In vivo gene overexpression or knockdown was achieved by AAV2/9 vectors. AAV2/9 vectors carrying DKK3 (pHBAAV2/9-CMV-m-DKK3-3xflag-T2A-mcherry) or empty vector with a cytomegalovirus (CMV) promoter, and AAV2/9 vectors carrying shDKK3(pHBAAV2/9-SM22α-mir30-m-DKK3-3xflag-T2A-mcherry) or empty vector with an SM22α promoter were manufactured by Hanbio (Beijing, China). ShDKK3 sequence: CCTGGCAAGCTTACCTCCCAACTAT. AAV2/9 vectors (1.5 × 10^11^ vg per mice) were delivered by tail vein injection to 8-week-old male mice. DKK3 overexpression or knockdown was verified by qPCR. Four weeks after injection, these mice were treated with 1,000 ng/(kg × min) Ang II for 4 weeks.

ATF6 agonist AA147 was dissolved in dimethyl sulfoxide (DMSO) to prepare a 5 mg/ml stock solution. Then, the storage solution was diluted with physiological saline to prepare a working solution of 0.5 mg/ml. AA147 (2 mg/kg) was administered intraperitoneally 1 d before and within 5 d after implantation of Ang II micropump.

### Bone marrow transplantation

The bone marrow of 8-week-old *Dkk3*^+/+^ *Apoe*^−/−^ and *Dkk3*^−/−^ *Apoe*^−/−^ mice was transplanted into the even-aged male recipients after lethally (8.5 Gy) γ-irradiation. Specifically, 4 h after irradiation, 1 × 10^7^ bone marrow cells flushed from the femurs of *Dkk3*^+/+^*Apoe*^−/−^ and *Dkk3*^−/−^ *Apoe*^−/−^ mice were injected by tail vein in each recipient mouse. After 4 weeks, these mice were treated with 1,000 ng/(kg × min) Ang II for 4 weeks.

### Vascular ultrasonic studies

Vascular ultrasonography was performed by Vevo 2100 console (Visual Sonics Vevo 2100, FUJIFILM, Bothell, WA, USA) to measure the abdominal aortic diameter in mice.

An MS 550D transducer (40 MHz center frequency, 7 mm focal length) was employed to acquire the image of the abdominal aorta. The ultrasound measurement was located at the suprarenal segment of the abdominal aorta, with visualization of the celiac trunk and superior mesenteric artery branches of the abdominal aorta. The maximal diameter of the external abdominal aortas was quantified using ultrasonic imaging analysis software.​ AAA was defined as an increase of at least 50% of normal in the maximal external diameters.

### Histological and immunofluorescence analysis

Mice are euthanized by CO_2_ inhalation, and then blood is collected by heart puncture. After perfusion with physiological saline, the abdominal aortas from experimental mice were immersion-fixed in 4% paraformaldehyde, embedded in optimal cutting temperature compound (OCT) compound, and sectioned at 7-μm thickness, and frozen aortic sections were stained with hematoxylin (RY-ICH001a, Roby, Beijing, China) and eosin (RY-ICH002a, Roby, Beijing, China) (H&E). EVG staining was conducted by the Elastic Stain Kit (Ab150667, Abcam, Cambridge, MA, USA). The number of elastin ruptures in aortic sections was quantified as follows: For each mouse, at least 3 aortic sections were analyzed, and the maximum count value was recorded.

After fixation in 4% paraformaldehyde (10 min) and membrane permeabilization using 0.1% Triton X-100 (10 min), aortic cryosections were incubated in blocking buffer with 3% bovine serum albumin (0332-100g, Amresco, Iowa, USA) at room temperature for 1 h. The samples were incubated with primary antibodies (Table S4) at 4 °C overnight and with secondary antibodies (Table S4) for 1 h at room temperature and mounted with 4′,6-diamidino-2-phenylindole (DAPI) (H-1200, Vector Laboratories, Beijing, China). DKK3 detection on aortas from AA patients or healthy donors was performed using DAB Detection Kit (gK600505, Gene Tech, Shanghai, China). Images were obtained through Leica TCS SP8 confocal fluorescent microscope (Germany) and Olympus BX53 fluorescent microscope (Japan).

### Cell culture and treatments

Mouse primary VSMCs were isolated from 8- to 10-week-old female *Dkk3^+/+^Apoe*^−/−^ and *Dkk3*^−/−^*Apoe*^−/−^ mice. The aortas were placed in 1.25 mg/ml collagenase type II (LS004174, Worthington, Franklin, OH, USA) for 0.5 h, and the adventitia were removed and placed in a digestive enzyme solution supplemented with collagenase type II (1.25 mg/ml) and elastase (E0258, Sigma, St. Louis, MO, USA) (50 μg/ml). Following digestion, the isolated cells were grown in smooth muscle cell medium (SMCM) media (1101, ScienCell, San Diego, CA, USA). Cell cultures within the passage range of 3 to 8 were utilized for in vitro experimentation. VSMCs were treated with 1 μM Ang II (ab120183, Abcam, Cambridge, MA, USA) for 24 h after starvation [2% fetal bovine serum (FBS)] for 12 h.

HASMC (CRL-1999, American Type Culture Collection, USA) was cultured in SMCM media. The siRNA against human DKK3 was designed and synthesized by Hanbio (Beijing, China). As the commercial protocol, siRNA (50 nM) (Hanbio, Beijing, China) transfection was applied in HASMC by RNAiMax (Invitrogen, CA, USA) reagent. HASMCs were treated with 1 μM Ang II or 3 μM SIS3 (HY-13013, MCE, CA, USA) for 24 h after starvation (2% FBS) for 12 h after transfection.

### Cell contraction assay

About 1 × 10^6^ cells were resuspended in 3.0 mg/ml type I collagen solution (Cell Biolabs Inc.) according to the manufacturer’s instructions. The cell–collagen suspension (500 μl) was plated in 24-well culture dishes and incubated at 37 °C for 1 h. SMCM (1 ml) supplemented with 10% FBS was added to each collagen gel lattice. After 2 d, the cells were treated with Ang II (1 μM) and gently released from the collagen gels on the sides of the culture plates. Photos were taken at 0 h, and after 24 h, the collagen gel size change was measured using ImageJ software.

### Real-time quantitative PCR

Total RNA was extracted using TRIzol reagent (15596018, Invitrogen, Carlsbad, CA, USA) from homogenized tissues or cultured cells. Retro-transcription in cDNA was generated by GoScript Reverse Transcription System (A5001, Promega, Mannheim, Germany). ​Quantitative real-time PCR analysis employed the SYBR Green Kit (420A, Takara, Shiga, Japan) using CFX Connect Real-Time System (Bio-Rad, Hercules, CA, USA). *Actb* mRNA was used as an endogenous control. The target gene mRNA levels were quantified as fold change in comparison with the control samples. Primers are listed (Table S5).

### Western blot

Protein was extracted independently from aortas and whole cells using RIPA Lysis Buffer (C1053, Applygen, Beijing, China), added with 1% protease inhibitors (78429, Thermo, MA, USA) and phosphatase inhibitors (78426, Thermo, MA, USA). Pierce BCA Protein Assay Kit (23209, Thermo Fisher Scientific, Waltham, MA, USA) was employed for protein concentration assessment. Forty micrograms of protein per sample was resolved via sodium dodecyl sulfate–polyacrylamide gel electrophoresis (SDS-PAGE) and electroblotted onto polyvinylidene difluoride membranes. Membranes were incubated with primary antibodies (Table S4) overnight at 4 °C. Secondary antibodies (Table S4) were incubated for 1 h at room temperature and underwent triplicate tris-buffered saline with Tween 20 (TBST) washes, followed by chemiluminescence detection using HRP Substrate Kit (Millipore, Bedford, MA, USA). Band densitometry was quantified via ImageJ (V1.8.0).

### Co-immunoprecipitation

Cells were lysed by Magnetic Co-Immunoprecipitation (Co-IP) Kit (Bes3011, BersinBio, China). Protein samples were incubated with DKK3 antibodies or immunoglobulin G (IgG) control overnight at 4 °C, followed by incubation with protein A/G beads. Complexes were stringently washed, eluted, and analyzed by SDS-PAGE followed by IB with ATF6 antibody followed by IB with DKK3 antibody.

### ChIP-qPCR

About 2 × 10^7^ cells of HASMC were collected for ChIP-qPCR. Nuclei were lysed, and chromatin was sheared to 200- to 1,000-base pair (bp) fragments via sonication after crosslinking protein–DNA interactions using 1% formaldehyde. Precleared lysates were immunoprecipitated overnight at 4 °C with DKK3 antibody or IgG control, followed by incubation with protein A/G beads. Complexes were stringently washed, eluted, and reverse-crosslinked. DNA was purified using DNA Purification Kit (28104, Qiagen, Germany) and then quantified by qPCR using SYBR Green qPCR Master Mix (QPK-201T, TOYOBO, Japan). The primer sequences for ATF6 in ChIP are as follows: forward primer (AGGGTAGACTCGCTTGGACT); reverse primer (GGGAAGACACGCAGACATCA).

### RNA-seq preparation and data analysis

For RNA sequencing, aortas were collected from *Dkk3^+/+^Apoe*^−/−^ and *Dkk3*^−/−^*Apoe*^−/−^ mice treated with saline or Ang II (*n* = 3 to 4 mice per group). After rapid freezing in liquid nitrogen, samples were transported using dry ice to Cnkingbio Biotechnology Corporation for total RNA extraction and subsequent RNA-seq analysis.

RNA-seq libraries were constructed with NEBNext UltraTM RNA Library Prep Kit for Illumina (NEB, USA), followed by sample indexing and cluster generation on cBot System (TruSeq PE Cluster Kit v3, Illumina) per manufacturer’s protocol. After cluster generation, the library preparations were sequenced on an Illumina Novaseq platform and 150-bp paired-end reads were generated. R package heatmap (session: 1.0.12) was used to perform hierarchical clustering based on differentially expressed mRNA. GO enrichment analysis characterized core functional attributes of differentially expressed mRNAs, and statistical significance was defined as *P* < 0.01. KEGG database analysis was utilized to identify significant signaling pathways of DEGs. ​Statistical significance was defined as *P* < 0.05.

### scRNA-seq data analysis

Data of scRNA-seq were accessed from the public GEO database (the data of human aortic tissues were from GSE155468, the data of mouse AAA for 7 and 14 d were from GSE152583, the data of mouse AAA for 28 d were from GSE221789). scRNA-seq data were processed with Seurat package (version 4.4.0), following a standardized workflow. This included normalization, feature selection, and principal components analysis (PCA)-based highly variable gene detection. Cells with fewer than 500 unique molecular identifiers (UMIs), fewer than 200 detected genes, or over 10% mitochondrial RNA content were excluded. Genes expressed in fewer than 5 cells were also removed. The filtered expression matrix was normalized based on the total number of UMIs per cell and log_2_-transformed. Highly variable genes were used to scale the data for PCA, and the first 15 principal components with 500 nearest neighbors were utilized to generate UMAP embeddings. Cell typing utilized established cell markers.

### Ligand–receptor cellular communication analysis

Cell–cell communication analysis was conducted through the CellChat computational framework, initiating with the identification of differentially expressed signaling genes (*P* < 0.05) across single-cell clusters to delineate specific cellular interactions. Subsequently, social network analysis extracted multidimensional signaling components—including cellular sources, regulatory nodes, effector targets, molecular mediators, and high-order communication patterns. Finally, ligand–receptor pair associations with cell populations were quantitatively mapped and visualized via alluvial diagramming techniques.​

### Identification of DEGs from microarray data

Data from microarray were accessed from the public GEO database (GSE57691). DEGs from each microarray were obtained with GEO2R online tool (https://www.ncbi.nlm.nih.gov/geo/geo2r/) [74], and all DEGs were identified as *P* value < 0.05 and fold change over 1.2 or less than 0.83 [|log_2_ (fold change)| ≥ 0.263].

### Statistical analysis

All data points are from distinct samples. The data of mice are expressed as the mean ± SEM, and the data of human plasma DKK3 level are expressed as the mean ± SD. Statistical analyses were conducted with GraphPad Prism 8.0 software (GraphPad Software, San Diego, CA, USA) and SPSS 24.0 (IBM, Armonk, NY, USA). For 2 groups of comparisons, the unpaired 2-tailed Student’s *t* test was applied to normal distribution, and the Mann–Whitney test was used when the data followed a non-normal distribution. Differences among multiple groups with multivariable data were analyzed by 2-way analysis of variance (ANOVA) followed by Bonferroni’s post hoc test. AAA incidence and rupture rates were analyzed via the Fisher exact test. Ang II-infused mice survival was analyzed via Kaplan–Meier survival curve. The significance threshold was set at *P* < 0.05.​

## Data Availability

The data of this study are available from the corresponding authors on reasonable request.
