# Supplementary material for: Elevated Dickkopf 3 Promotes Abdominal Aortic Aneurysm Formation via Modulated Phenotype Switch of Vascular Smooth Muscle Cells
Source: Research (Wash D C). 2025 Sep 11;8:0873. doi: 10.34133/research.0873 (PMC12423507; doi:10.34133/research.0873)
Supplement: Supplementary 1 — Figs. S1 to S11 Tables S1 to S5 [file research.0873.f1.docx]

**Supplemental Materials**

**Elevated Dickkopf 3 Promotes Abdominal Aortic Aneurysm Formation via Modulated Phenotype Switch of Vascular Smooth Muscle Cells**

Xuejie Cao ^1#^, Jinmeng Jia ^2#^, Qiuyue Gao ^1#^, Jiaping Tao ^1^, Ming Wei ^1^, Yanting Song ^3^, Hong Wu ^4^, Shiyu Jiao^1^, Xinxin Zhu ^1^, Xuegong Zhang ^2,5^, Yi Fu ^6^, Yuan Wang^7^, Jie Du ^7^, Qingbo Xu ^4*^, Aijuan Qu ^1*^ and Baoqi Yu ^1*^

1 Department of Physiology and Pathophysiology, School of Basic Medical Sciences, Capital Medical University, Key Laboratory of Remodeling-Related Cardiovascular Diseases, Ministry of Education, Laboratory for Clinical Medicine，Capital Medical University, Beijing 100069, China;

2 Bioinformatics Division of BRNIST and Department of Automation, MOE Key Lab of Bioinformatics, Tsinghua University, Beijing, 100084, China

3 Department of Pathology, Beijing Anzhen Hospital Affiliated to Capital Medical University, Beijing, 100029, China

4 Department of Cardiology, The First Affiliated Hospital, Zhejiang University School of Medicine, Hangzhou, 310016, China

5 School of Life Sciences, Center for Synthetic and Systems Biology, Tsinghua University, Beijing 100084, China

6 Department of Physiology and Pathophysiology, School of Basic Medical Sciences, Peking University, Beijing, 100191, China

7 Beijing Anzhen Hospital of Capital Medical University and Beijing Institute of Heart Lung and Blood Vessel Diseases, Beijing, 100029, China

X. Cao and J. Jia and Q.Gao contributed equally.

(Running title: DKK3 promotes AAA development)

**Supplemental Materials ：**

Figs. S1 to S11

Tables S1 to S5

**Supplemental Figures**

Fig. S1


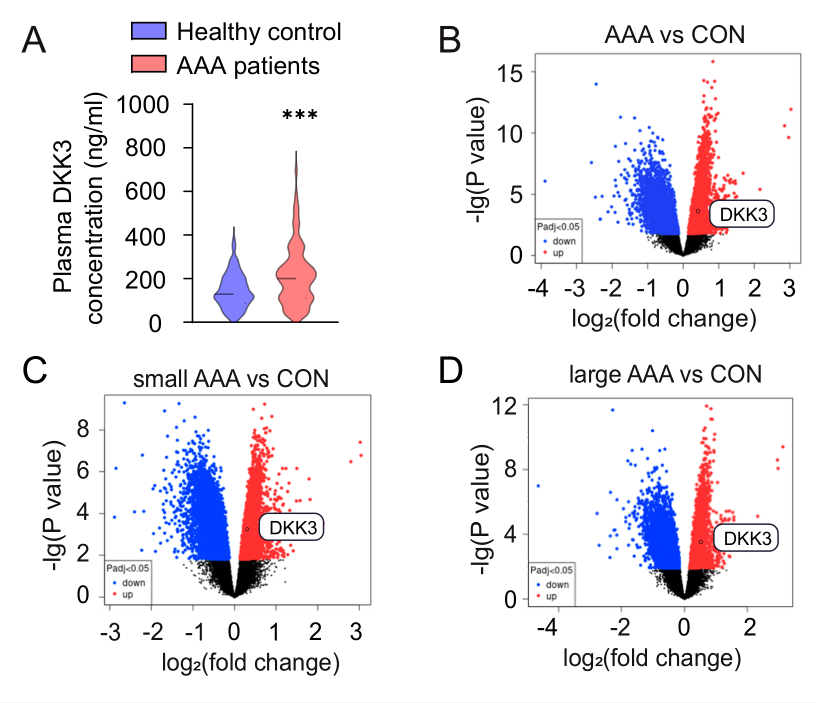


**Fig. S1.** DKK3 is significantly increased in development of abdominal aortic aneurysm (AAA).

(A) Plasma DKK3 levels were measured by ELISA in healthy volunteers (n=100) and AAA patients (n=100). ****P*<0.001, unpaired Student’s t-test. The microarray database GSE57691 was analyzed by GEO2R, the results showed DEGs volcano map between (B) AAA patients (n=49) and control (n=10) (C) patients with small AAA (n=20) and control(n=10) (D) patients with large AAA(n=29) and control(n=10); compared with the control, red dots represent upregulated genes, black dots represent genes with no significant difference, and blue dots represent downregulated genes in AAA patients.

Fig. S2


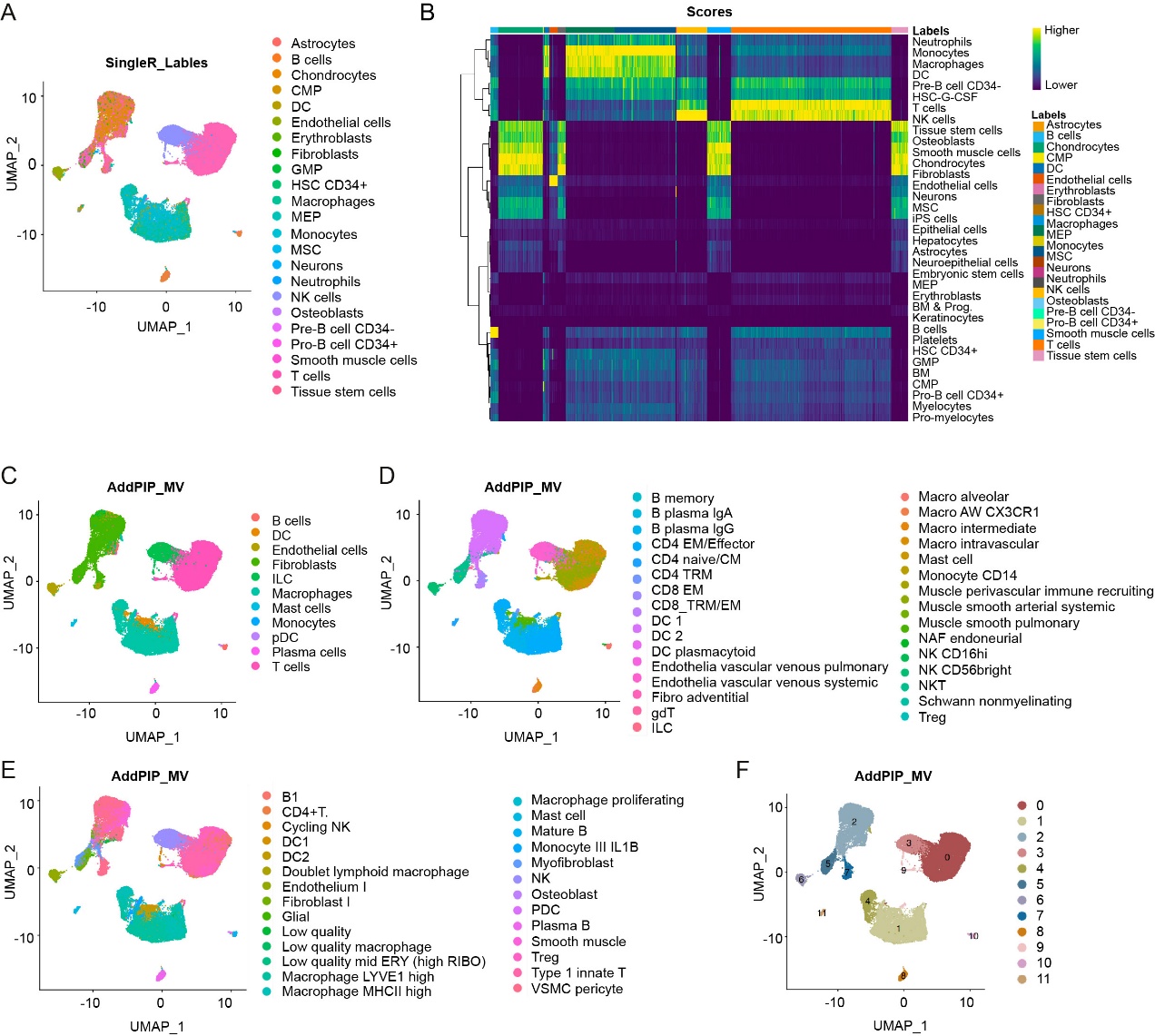


**Fig. S2.** The results of SingleR annotation and CellTypist annotation.

Clustering of GSE155468 single cells for identifying cell clusters. (A) The UMAP map of SingleR cell annotation results. (B) The Heatmap of SingleR annotation results. The cell annotation results of the default model (C) Immune_All_Low.pkl, as well as the model with strong correlation with vascular SMC (D) Cells_Lung_Airway.pkl, and (E) Pan_Fetal_Human.pkl. (F) UMAP plot of all the major cell types based on the annotation results of CellTypist and SingleR.

Fig. S3


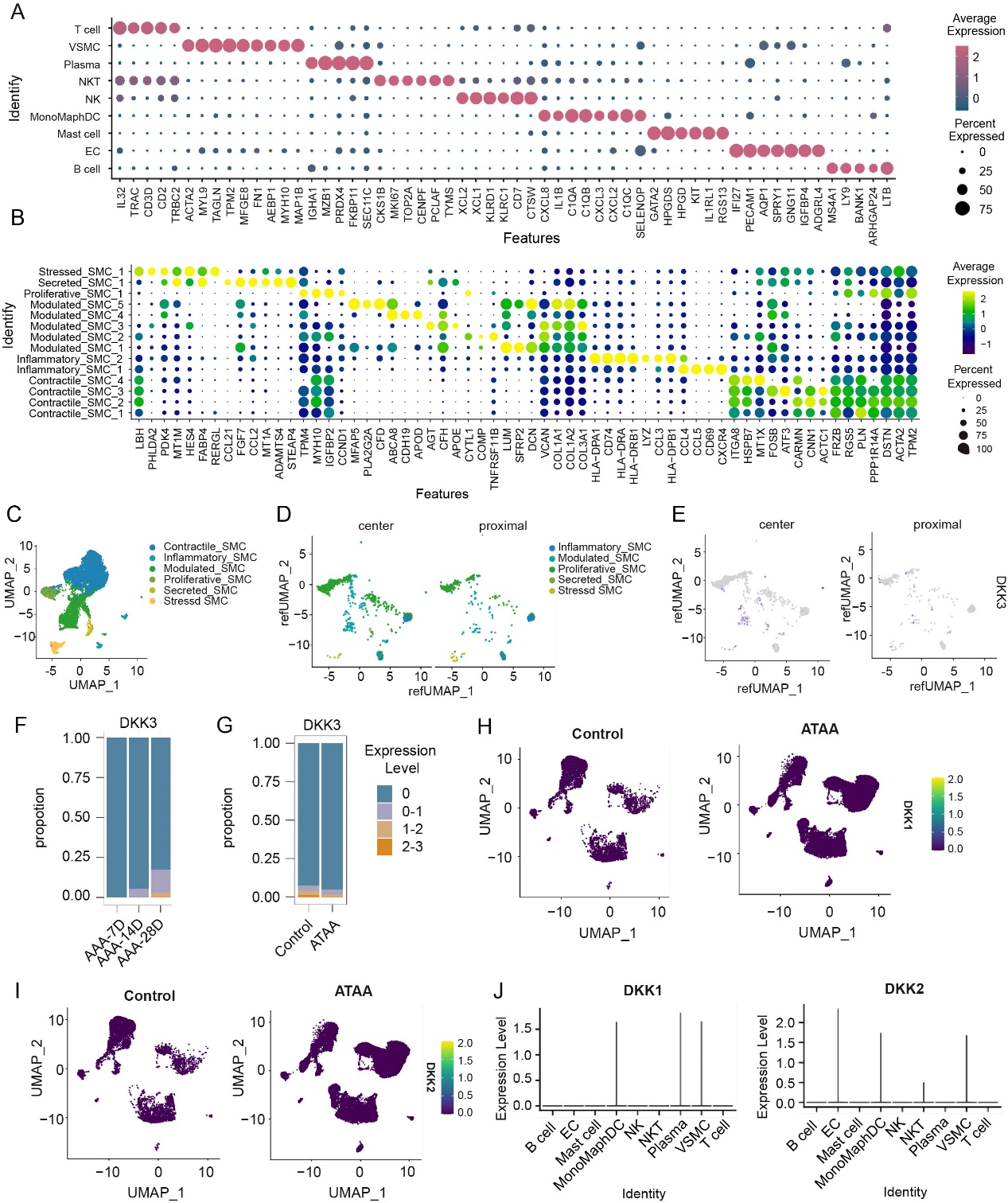


**Fig. S3.** DKK3 expression is increased in the VSMCs of aortic aneurysm patients.

Identification of cell clusters in AA patients by scRNA-seq analysis of a public dataset GSE155468. (A) Mean expression of selected genes in the major cell types. (B) The highest specificity gene markers for each VSMC subpopulation. (C) UMAP plot of VSMC subpopulations in aortic tissue of AAA and control in datasets GSE224587. Identification of cell clusters and key factors in AA patients by scRNA-seq analysis of a public dataset GSE224587. (D) UMAP plot of VSMC subpopulations of AAA center and AAA proximal. (E) Feature plots displaying the single-cell gene expression of DKK3 across cell clusters. Identification of key factors in AAA mouse model by scRNA-seq analysis of public datasets GSE152583 and GSE 221789. (F) The stacked bar plot represents the proportion of *Dkk3* in ECs in the AAA mouse model for 7D (n=5), 14D (n=5), 28D (n=12). (G) The stacked bar plot represents the proportion of *Dkk3* in ECs in aorta ATAA patients (n=8) and healthy control (n=3). Feature plots displaying the single-cell gene expression of (H) DKK1 and (I) DKK2 in the major cell types. (J) Violin plot of the differential expression of DKK1 and DKK2 in different kinds of cells in the human aorta by scRNA-seq analysis of a public dataset GSE155468.

Fig. S4


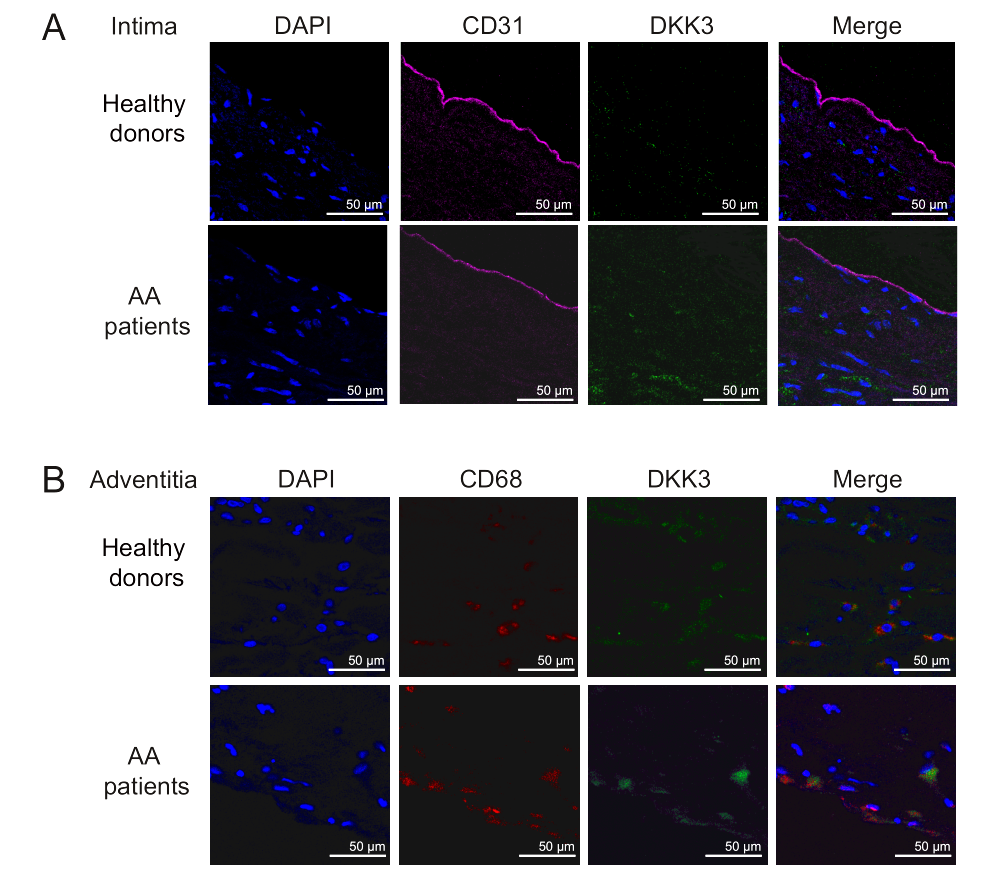


**Fig. S4.** DKK3 is significantly increased in development of abdominal aortic aneurysm (AAA).

Immunofluorescence staining of (A) DAPI (blue), CD31 (pink) and DKK3 (green) in human normal aorta(n=5) and AA tissues (n=6) and (B) DAPI (blue), CD68 (red) and DKK3 (green) in human normal aorta (n=5) and AA tissues (n=3).

Fig. S5

**
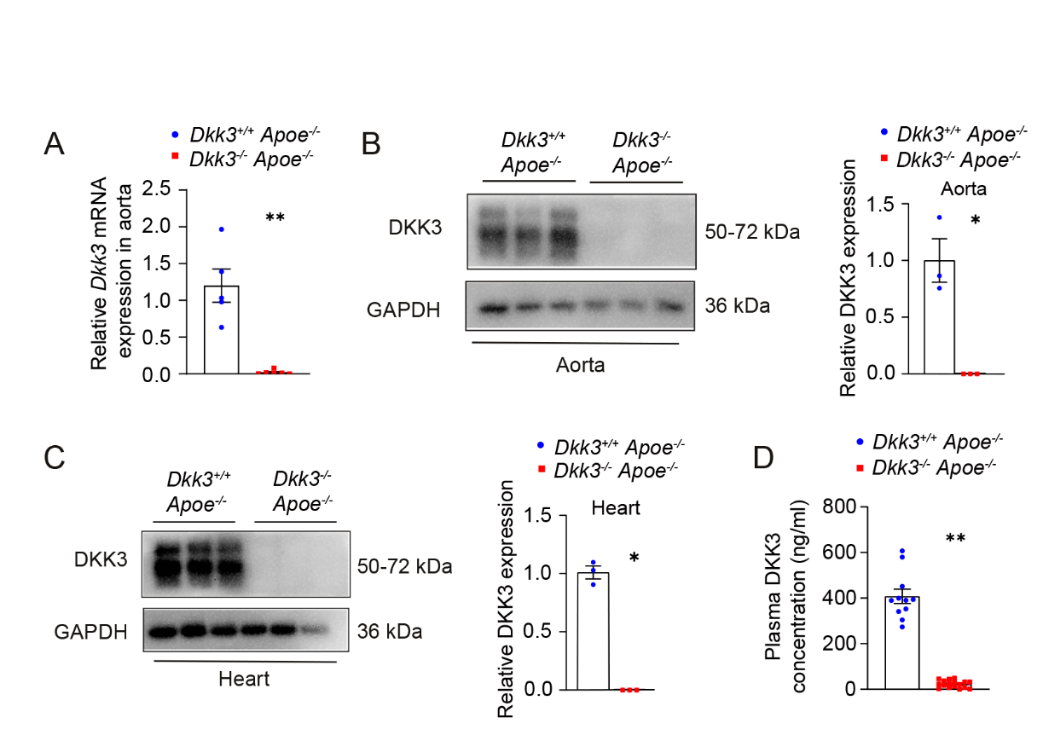
**

**Fig. S5.** Identification of DKK3 knockout.

(A) *Dkk3* expression in aorta was examined by qPCR in *Dkk3*^+/+^*Apoe*^-/-^ mice and *Dkk3^-/-^Apoe^-/-^* mice (n=5-6). The DKK3 level was detected by Western Blot in the (B) aorta and (C) heart of *Dkk3*^+/+^*Apoe*^-/-^ mice and *Dkk3^-/-^Apoe^-/-^* mice (n=3). Quantification of Western Blot analysis for DKK3, normalized to GAPDH (n=3). (D) The plasma DKK3 level detected by ELISA in *Dkk3*^+/+^*Apoe*^-/-^ mice (n=11) and *Dkk3*^-/-^*Apoe*^-/-^ mice (n=17). **p* < 0.05, ***p* < 0.01, unpaired Student’s t-test for A, B, C, D.

Fig. S6


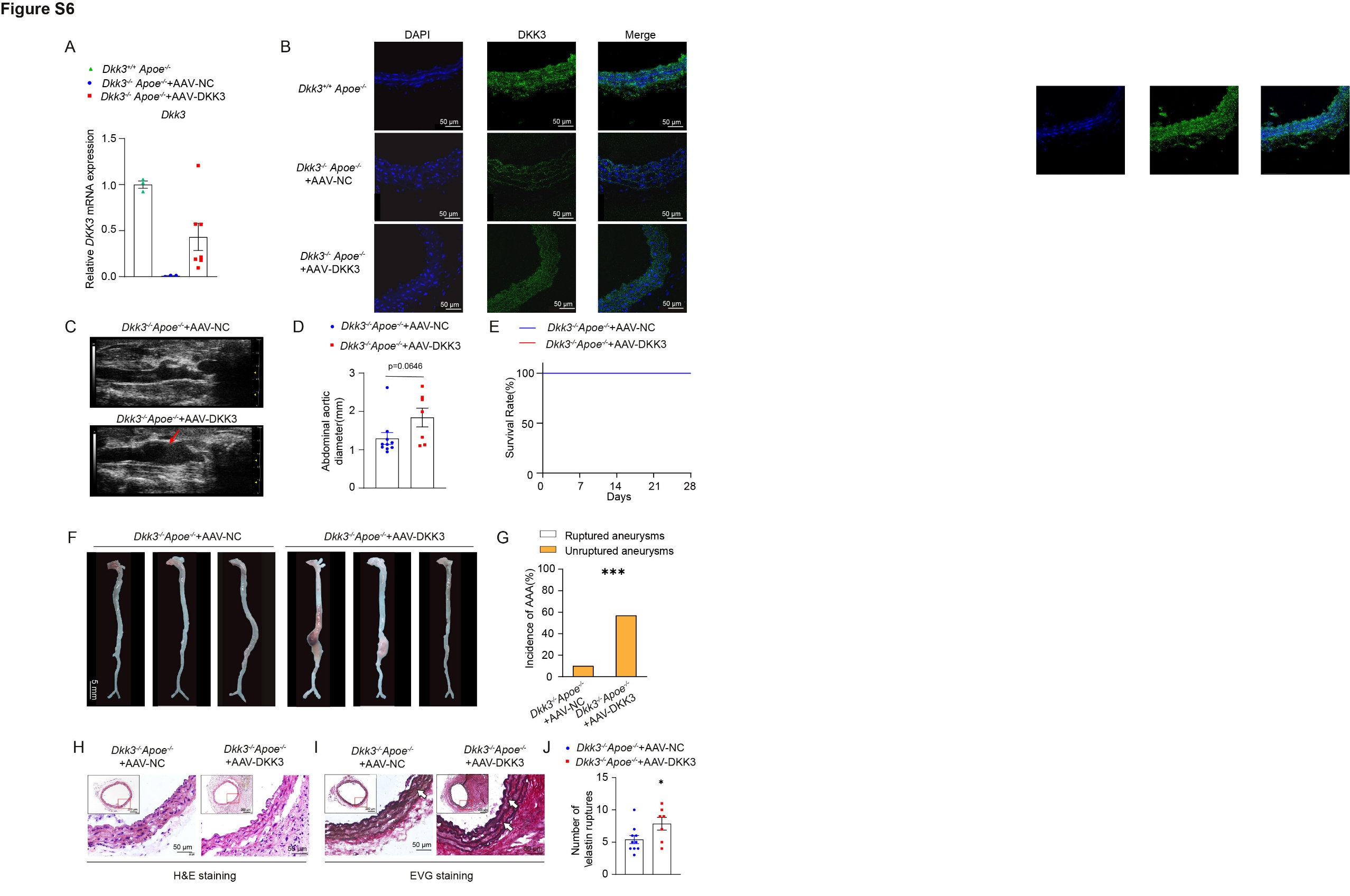


**Fig. S6.** Supplementing DKK3 levels with AAV-DKK3 accelerated AAA development.

(A) The *Dkk3* levels in the aorta of *Dkk3*^+/+^*Apoe*^-/-^ mice, *Dkk3*^-/-^*Apoe*^-/-^ mice injected with AAV-NC and *Dkk3*^-/-^*Apoe*^-/-^ mice injected with AAV- DKK3 were detected by qPCR(n=3-7). (B) Immunofluorescence staining of DAPI (blue) and DKK3(green) in the abdominal aorta of *Dkk3*^+/+^*Apoe*^-/-^ mice, *Dkk3*^-/-^*Apoe*^-/-^ mice injected with AAV-NC and *Dkk3*^-/-^*Apoe*^-/-^ mice injected with AAV- DKK3. (C) B-mode ultrasound of abdominal aorta in each group (*Dkk3*^-/-^*Apoe*^-/-^ +AAV-NC and *Dkk3*^-/-^*Apoe*^-/-^ +AAV-DKK3) treated with Ang Ⅱ. Arrows indicate the dilated aorta. (D) Statistical analysis of maximal abdominal aortic diameter in each group treated by Ang Ⅱ separately (n=7-10), unpaired Student’s t-test. (E) The survival rates of the two groups, Gehan-Breslow-Wilcoxon test. (F) Representative photos of the aorta of the 2 groups of mice. (G) The incidence of AAA in the two groups of mice treated by Ang Ⅱ separately (n=7-10). ****P*<0.001, Chi-square test. Representative (H) H&E and (I) EVG stainings of the abdominal aortas in each group. Arrows indicate elastin degradation areas. (J) Quantification of the number of elastin breaks per vessel (n=7-10), **p* < 0.05, unpaired Student’s t-test.

Fig. S7


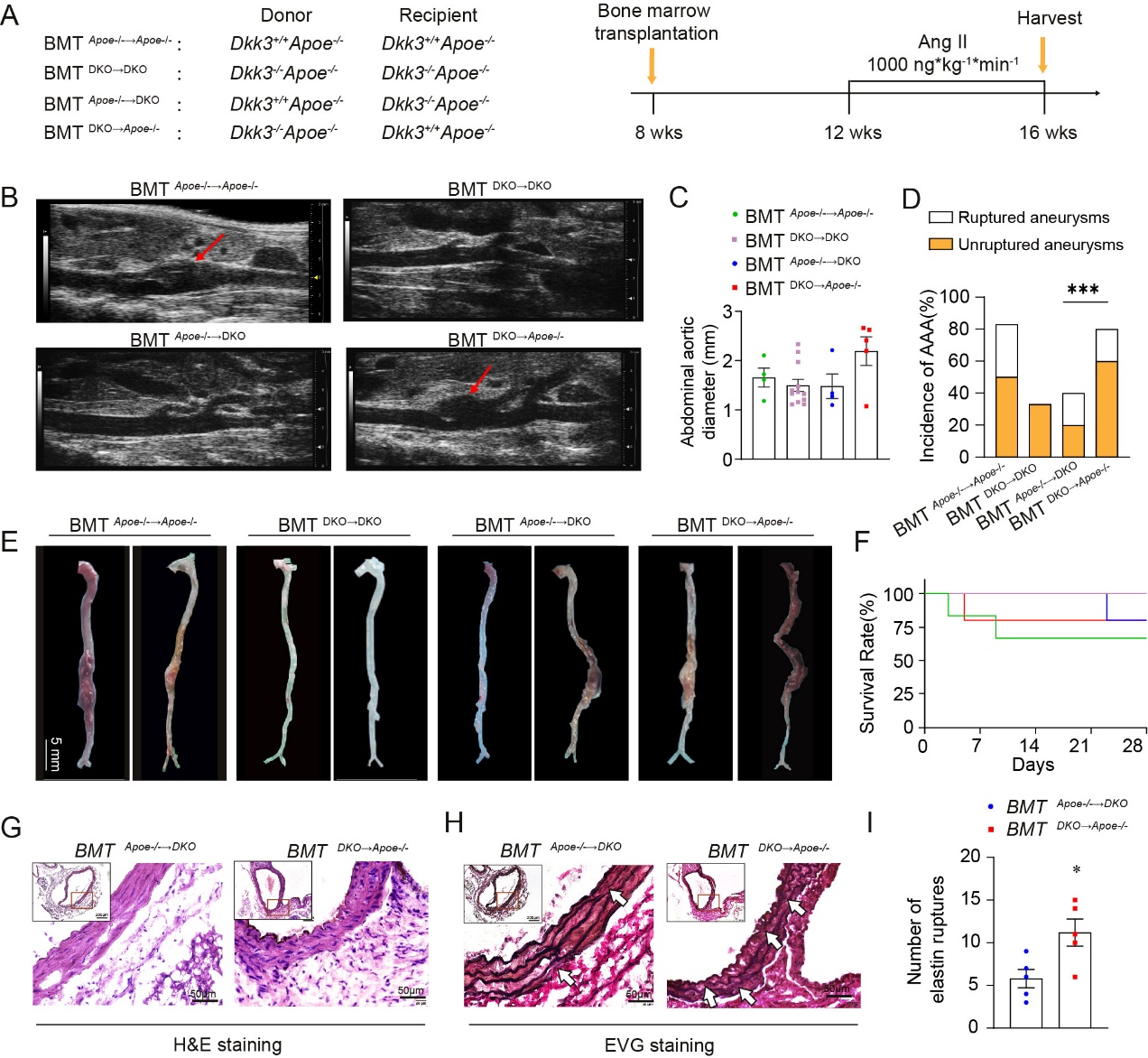


**Fig. S7.** Deletion of non-bone marrow-derived DKK3 significantly inhibited AAA development.

(A) Schematic diagram of bone marrow transplantation experiments. (B) B-mode ultrasound of abdominal aorta in bone marrow transplanted mice. Arrows indicate the dilated aorta. (C) Statistical analysis of maximal abdominal aortic diameter in each group (BMT *^Apoe-/-→Apoe-/-^* mice (n=4), BMT ^DKO→DKO^ mice (n=12), BMT *^Apoe-/-^*^→DKO^ mice (n=4) and BMT ^DKO^*^→Apoe-/-^* mice (n=5) treated with Ang Ⅱ, unpaired Student’s t-test. (D) The incidence rate of AAA in bone marrow transplanted mice treated with Ang Ⅱ. ****P*<0.001, Chi-square test. (E) Representative photos of abdominal aorta in the bone marrow transplanted mice treated with Ang Ⅱ separately. (F) The survival rate of bone marrow transplanted mice treated with Ang Ⅱ, Gehan-Breslow-Wilcoxon test. Representative (G) H&E and (H) EVG staining of the abdominal aorta in BMT *^Apoe^*^-/-^*^→^*^DKO^ mice (n=5) and BMT ^DKO^*^→ Apoe^*^-/-^mice (n=5) treated with Ang Ⅱ. Arrows indicate elastin degradation areas. (I) Quantification of the number of elastin breaks per vessel (n=5). **P*<0.05, unpaired Student’s t-test.

Fig. S8

**
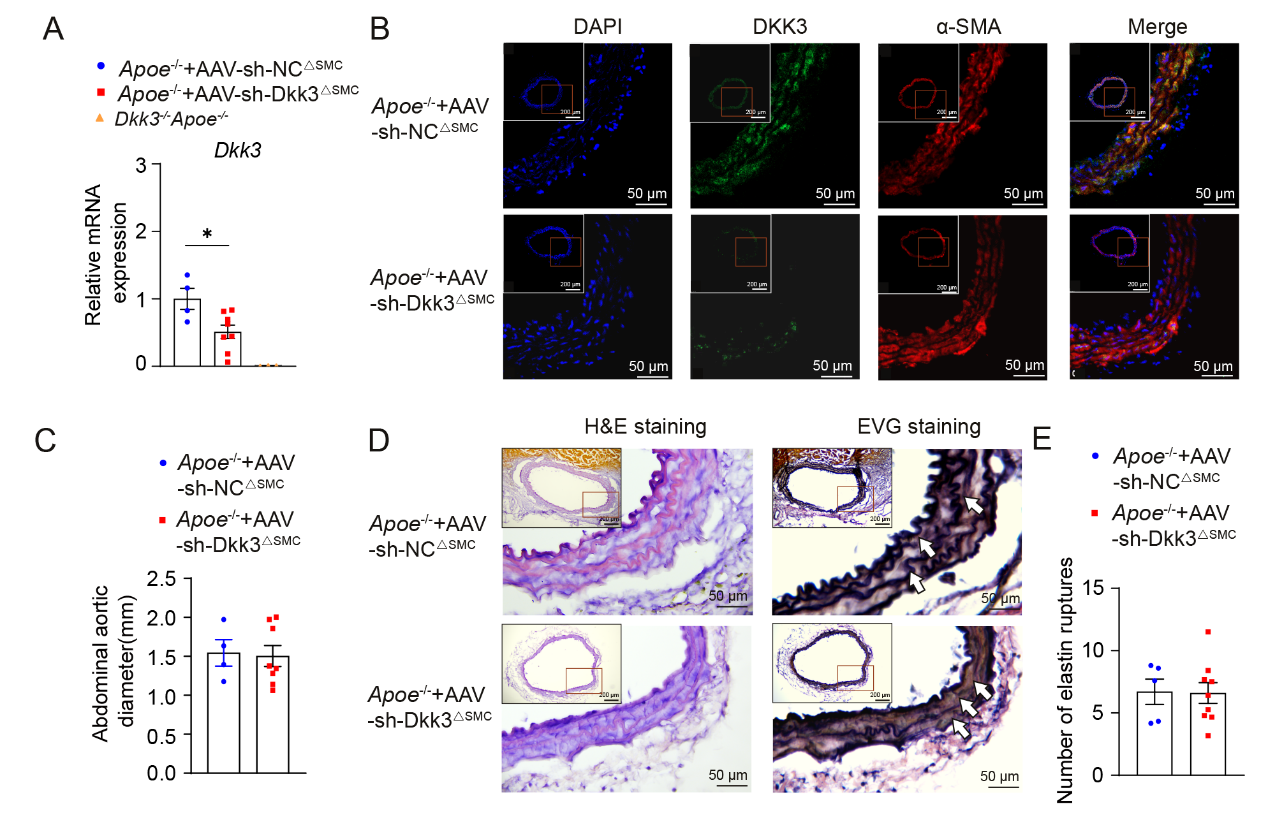
**

**Fig. S8.** The impact of VSMC-specific DKK3 knockdown on the development of AAA.

(A) The *Dkk3* levels in the aorta of each group were detected by qPCR (*Apoe*^-/-^ + AAV-sh-NC^∆SMC^, n = 4; *Apoe*^-/-^+ AAV-sh-Dkk3^∆SMC^, n = 8; *Dkk3*^-/-^*Apoe*^-/-^, n = 3), **p* < 0.05, unpaired Student’s t-test. (B) Immunofluorescence staining of DAPI (blue), DKK3(green) and α-SMA (red) in the abdominal aorta of the *Apoe*^-/-^+AAV-sh-NC^∆SMC^ mice and *Apoe*^-/-^+AAV-sh-Dkk3^∆SMC^ mice.

Fig. S9


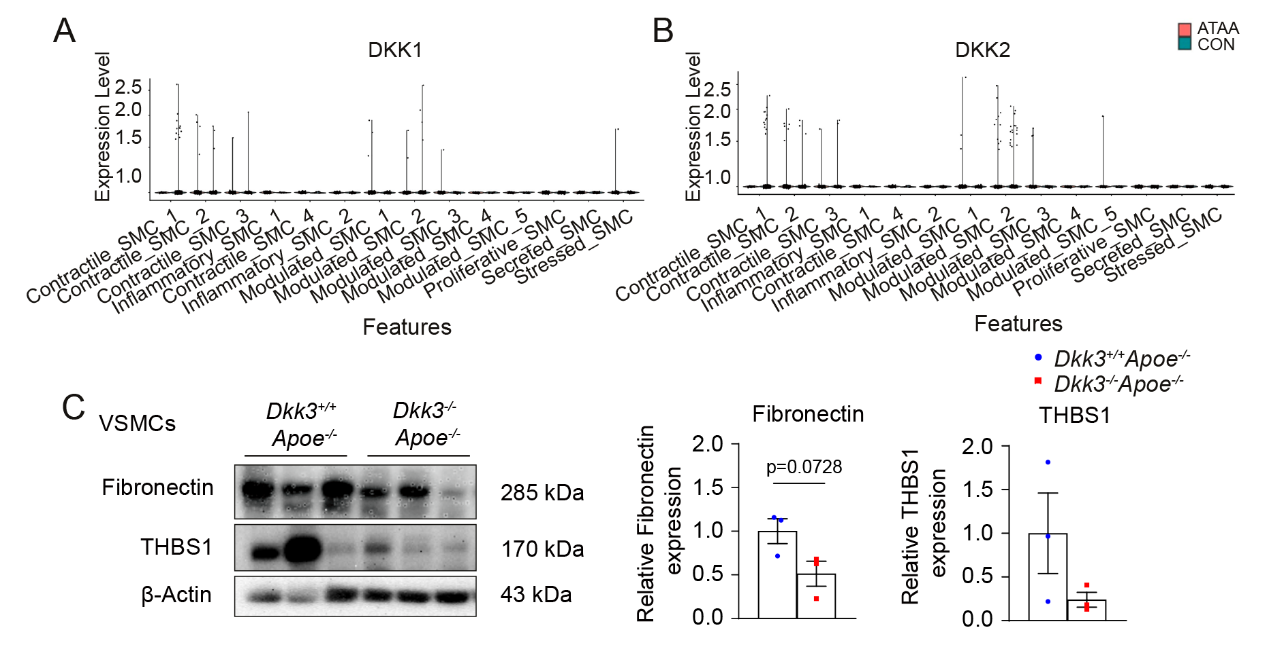


**Fig. S9.** DKK3 deficiency inhibited VSMC phenotypic transformation

Violin plot of the differential expression of (A) DKK1 and (B) DKK2 of VSMCs in the aorta of ATAA patients and healthy control. (C) The levels of VSMCs synthetic markers in *Dkk3*^+/+^*Apoe*^-/-^ and *Dkk3*^-/-^*Apoe*^-/-^ VSMCs were examined by Western Blot (n=3). unpaired Student’s t-test.

Fig. S10


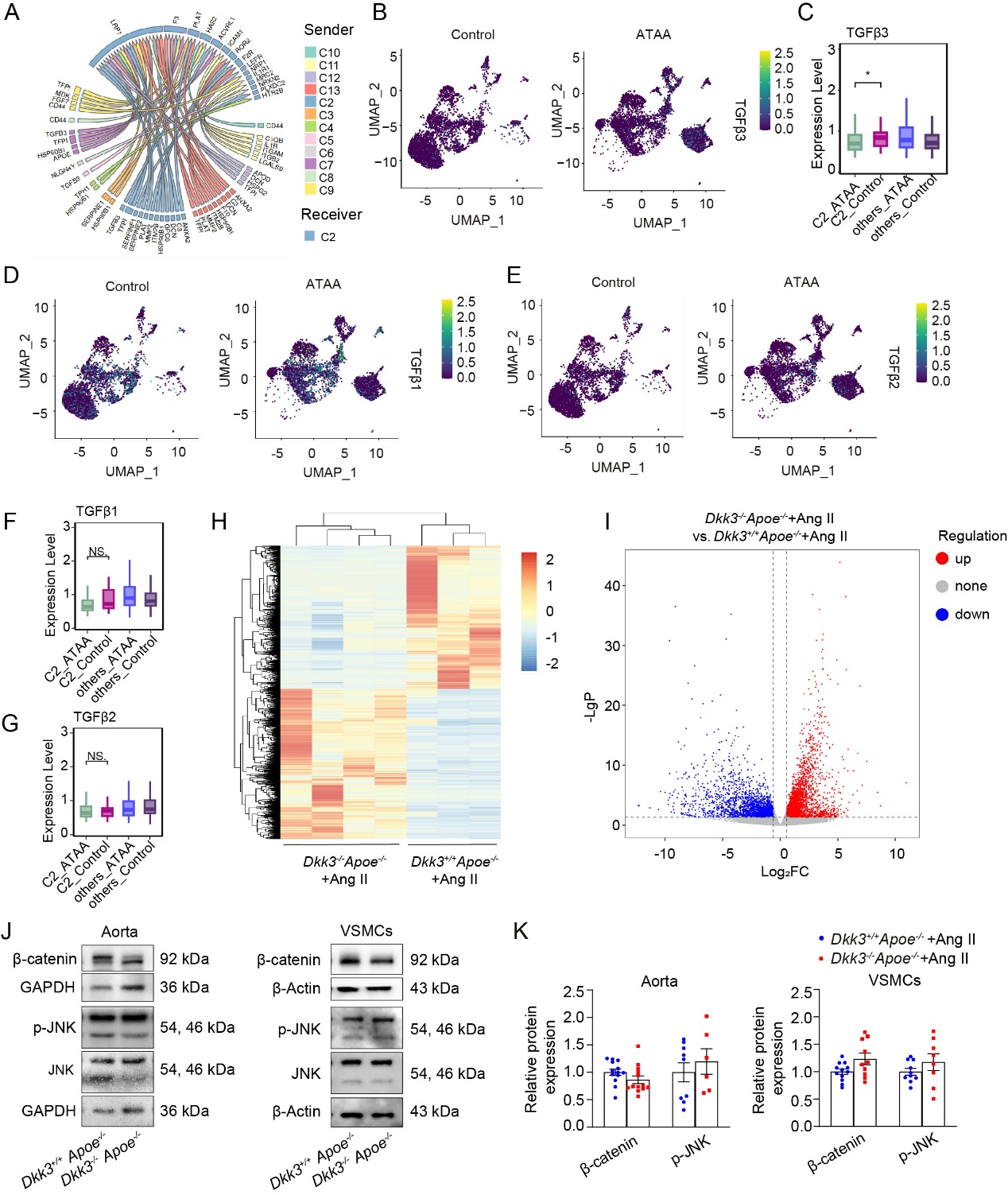


**Fig. S10.** DKK3 affects AAA process through TGF - β signaling pathway.

Identification of cell clusters in AA patients by scRNA-seq analysis of a public dataset GSE155468. (A) Chord Diagram emphasizing the network structure and sender-receiver relationships for each VSMC subpopulation. (B) Feature plots displaying the single-cell gene expression of TGFβ3 across cell clusters. (C) TGFβ3 expression in Modulated SMC1（cluster-C2）and other phenotype of VSMCs in Control and ATAA patients. Feature plots displaying the single-cell gene expression of (D) TGFβ1 and (E) TGFβ2 across cell clusters. Expression of (F) TGFβ1 and (G) TGFβ2 in Modulated SMC1 (cluster-C2) and other phenotype of VSMCs in Control and ATAA patients. (H) Heatmap of DEGs in the aortas of *Dkk3*^-/-^*Apoe*^-/-^ and *Dkk3*^+/+^*Apoe*^-/-^ mice treated with Ang Ⅱ (n=3-4 per group), FDR < 0.05. (I) Volcano Plot showing the upregulated and downregulated genes in the aortas of *Dkk3*^-/-^*Apoe*^-/-^ and *Dkk3*^+/+^*Apoe*^-/-^ mice treated with Ang Ⅱ. Compared with the *Dkk3*^+/+^*Apoe*^-/-^ mice, red dots represent upregulated genes, gray dots represent genes with no significant difference, and blue dots represent downregulated genes in *Dkk3*^-/-^*Apoe*^-/-^ mice. *P* < 0.05 and fold change > 2. (J) The β-catenin and p-JNK in aortas (n=6-13) and VSMCs (n=8-12) of *Dkk3^+/+^Apoe*^-/-^ and *Dkk3*^-/-^*Apoe*^-/-^ mice treated with Ang Ⅱ. (K) Quantification of Western Blot analysis for β-catenin normalized to GAPDH or β-actin and p-JNK normalized to JNK, unpaired Student’s t-test.

Figure S11


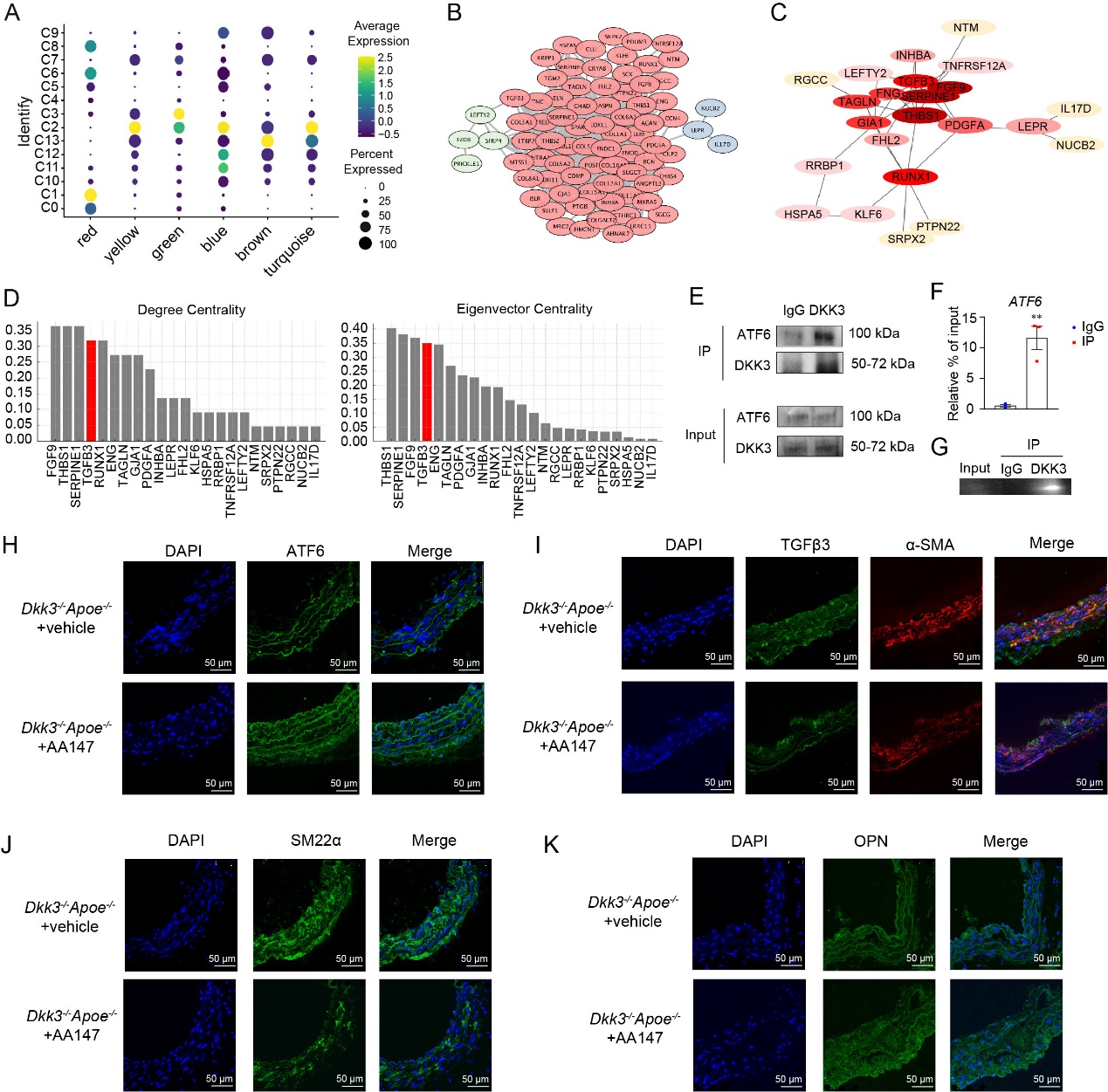


**Fig. S11.** DKK3 is proposed to regulate the TGF-β3 signaling pathway through ATF6.

Identification of cell clusters in AA patients by scRNA-seq analysis of a public dataset GSE155468. (A) Module Identification via high-resolution weighted gene co-expression network analysis (hdWGCNA). (B) STRING-Based Network Construction and Markov clustering algorithm (MCL) Clustering. (C) TGFB3 Submodule with HSPA5 (Centrality-Colored). (D) TGFB3 Submodule: Degree and Eigenvector Centrality. (E) Co-IP was used to verify the interaction between DKK3 and ATF6. Whole cell lysates from *Apoe*^-/-^ VSMCs were used for IP with DKK3 antibody followed by immunoblotting (IB) with ATF6 antibody followed by IB with DKK3 antibody. (F) ChIP-qPCR analysis of ATF6 promoters using DKK3 antibodies in HASMC. (G) C-PCR result confirmed the interaction between DKK3 and the promoter of ATF6. Immunofluorescence staining of (H) DAPI (blue), ATF6 (green), (I) DAPI (blue), TGFβ3 (green), α-SMA (red), (J) DAPI (blue), SM22α (green), (K) DAPI (blue), OPN (green) in the abdominal aorta of the *Dkk3*^-/-^*Apoe*^-/-^ +vehicle mice and *Dkk3^-/-^Apoe^-/-^*+AA147 mice.

**Supplemental Tables**

**Table S1**. **Clinical and biochemical characteristics of AAA patients and healthy volunteers included in the study**

| **Factors** | **AAA patients**  **(n=100)** | **Healthy volunteers**  **(n=100)** | ***P* value** |
| --- | --- | --- | --- |
| Gender (male) | 89 (89%) | 89 (89%) |  |
| Age (years) | 67.9 ± 10.8 | 67.48 ± 11.65 | 0.7690 |
| Smoking (n=160) | 45/100 (45%) | 15/60 (25%) | 0.011* |
| Hypertension (n=160) | 85 /100 (85%) | 24/60 (40%) | 0.007** |
| Diabetes mellitus (n=159) | 20/100 (20%) | 9/59 (15.25%) | 0.454 |
| Triglycerides (mg/dL) | 2.91 ± 13.69 | 1.66 ± 1.73 | 0.416 |
| Blood glucose  (mmol/L) | 6.88 ± 3.82 | 5.84 ± 1.56 | 0.111 |
| Homocysteine (μmol/L) | 17.15 ± 8.67 | 16.7 ± 6.71 | 0.063 |
| DKK3 (ng/mL) | 267 ± 317 | 168± 141 | 0.005^##^ |

All data are given as mean ± SD.

**P* < 0.05, ***P* < 0.01 by Chi-square test.

^##^ *P* < 0.01 by Student’s t-test.

**Table S2. Multivariate logistic regression analyses of plasma DKK3 levels and AAA**

| Model | OR (95% Cl) | P value |
| --- | --- | --- |
| Unadjusted Model | 1.003 (1.001-1.005) | 0.008 |
| Adjusted by gender, age, hypertension and smoking | 1.003 (1.000-1.006) | 0.085 |

**Table S3. PCR Primer Pairs**

| **Primer** | **Sequences (5’-3’)** |
| --- | --- |
| *Apoe* | P1: GCC TAG CCG AGG GAG AGC CG |
|  | P2: TGT GAC TTG GGA GCT CTG CAG C |
|  | P3: GCC GCC CCG ACT GCA TCT |
| *Dkk3* | P1: GAT AGC TTT CCG GGA CAC AC |
|  | P2: TCC ATC AGC TCC TCC ACC TCT |
|  | P3: TAA GTT GGG TAA CGC CAG GGT |

*Apoe*:

Homozygote= ~245 bp

Heterozygote = ~155 bp and ~245 bp

Wild type = ~155 bp

*Dkk3*:

Homozygote= ~199 bp

Heterozygote = ~199 bp and ~220 bp

Wild type = ~220 bp

**Table S4: Antibodies.**

| **Antibody** | **Manufacturer** | **Catalogue number** | **Dilution** |
| --- | --- | --- | --- |
| DKK3 | Abcam | ab186409 | 1:100 (immunofluorescence staining);  1:1000 (Western Blot) |
| DKK3 | R&D | AF948 | 1:100 (immunofluorescence staining) |
| SM22α | Abcam | ab14106 | 1:100 (immunofluorescence staining);  1:1000 (Western Blot) |
| MMP2 | Abcam | ab92536 | 1:100 (immunofluorescence staining);  1:1000 (Western Blot) |
| F4/80 | CST | 70076 | 1:100 (immunofluorescence staining) |
| α-SMA | CST | 48938s | 1:100 (immunofluorescence staining) |
| CD31 | R&D | AF3628 | 1:100 (immunofluorescence staining) |
| MMP9 | Santa Cruz | sc-393859 | 1:100 (immunofluorescence staining);  1:1000 (Western Blot) |
| OPN | Abcam | ab63856 | 1:100 (immunofluorescence staining);  1:1000 (Western Blot) |
| CD68 | Abcam | ab955 | 1:100 (immunofluorescence staining) |
| ATF6 | Proteintech | 24169-1-ΑP | 1:100 (immunofluorescence staining);  1:1000 (Western Blot) |
| ACTB | Proteintech | 66009-1-lg | 1:2000 (Western Blot) |
| GAPDH | Proteintech | 10494-1-AP | 1:2000 (Western Blot) |
| β-catenin | BD | 610153 | 1:1000 (Western Blot) |
| p-SAPK/JNK (Thr183/Tyr185) | CST | 4668s | 1:1000 (Western Blot) |
| SAPK/JNK | CST | 9252s | 1:1000 (Western Blot) |
| MYH 11 | Abcam | ab133567 | 1:1000 (Western Blot) |
| Calponin 1 | Abcam | ab46794 | 1:1000 (Western Blot) |
| Fibronectin | Abcam | ab2413 | 1:1000 (Western Blot) |
| THBS1 | CST | 37879s | 1:1000 (Western Blot) |
| Smad2/3 | CST | 8685s | 1:1000 (Western Blot) |
| p-Smad2 (Ser465/467)/Smad3 (Ser423/425) | CST | 8828s | 1:1000 (Western Blot) |
| TGFβR1 | Abcam | ab112095 | 1:1000 (Western Blot) |
| TGFβ2 | Santa Cruz | sc-374658 | 1:1000 (Western Blot) |
| TGFβ3 | Santa Cruz | sc-166833 | 1:1000 (Western Blot) |
| donkey anti-goat AF488 | Invitrogen | A11055 | 1:800 (immunofluorescence staining) |
| donkey anti-rabbit AF647 | BIOSS | bs-0295D-A647 | 1:200 (immunofluorescence staining) |
| goat anti-rabbit TRITC | ZSGB-BIO | ZF-0316 | 1:200 (immunofluorescence staining) |
| goat anti-rabbit FITC | ZSGB-BIO | ZF-0311 | 1:200 (immunofluorescence staining) |
| goat anti-mouse FITC | ZSGB-BIO | ZF-0312 | 1:200 (immunofluorescence staining) |
| goat anti-mouse IgG, HRP-linked Antibody | CST | 7074S | 1:2000 (Western Blot) |
| goat anti-rabbit IgG, HRP-linked Antibody | CST | 7076S | 1:2000 (Western Blot) |

**Table S5. Real-Time PCR Primer Pairs**

| **Primer** | **Sequences (5’-3’)** |
| --- | --- |
| *Mmp2*  (mouse) | F: TCAACGGTCGGGAATACAGC |
|  | R: CCATGGTAAACAAGGCTTCATGG |
| *Mmp9*  (mouse) | F: TGGTCTTCCCCAAAGACCTG |
|  | R: AGCGGTACAAGTATGCCTCTG |
| *Dkk3*  (mouse) | F: CAGCTCTCAACTACCCTCAGG |
|  | R: ACCTCAGAGGACGTTTTAGCA |
| *Actb*  (mouse) | F: TTCTTTGCAGCTCCTTCGTT |
|  | R: ATGGAGGGGAATACAGCCC |
| *Myh11*  (mouse) | F: GTGTGGTGGTCAACCCCTAC |
|  | R: GATGTGAGGCGGCATCTCAT |
| *Opn*  (mouse) | F: CACTCCAATCGTCCCTACAGT |
|  | R: CTGGAAACTCCTAGACTTTGACC |
| *Calponin 1*  (mouse) | F: GCACATTTTAACCGAGGTCCT |
|  | R: CTGATGGTCGTATTTCTGGGC |
| *Thbs1*  (mouse) | F: TTCTTACCCTTGACAACAACGTG |
|  | R: CCACAGATAGCTTGGAGGTCC |
| *Sm22a*  (mouse) | F: CTCTAATGGCTTTGGGCAGTTTG |
|  | R: TGCAGTTGGCTGTCTGTGAAGTC |
| *Tgfb1*  (mouse) | F: CCACCTGCAAGACCATCGAC |
|  | R: CTGGCGAGCCTTAGTTTGGAC |
| *Tgfb2*  (mouse) | F: TCGACATGGATCAGTTTATGCG |
|  | R: CCCTGGTACTGTTGTAGATGGA |
| *Tgfb3*  (mouse) | F: GGACTTCGGCCACATCAAGAA |
|  | R: TAGGGGACGTGGGTCATCAC |
| *Atf6*  (mouse) | F: GGAGCCACTGAAGGAAGATAAG |
|  | R: GTGCTGCTGGAAGCAATAAAG |
| *DKK3*  (human) | F: AGGACACGCAGCACAAATTG |
|  | R: CCAGTCTGGTTGTTGGTTATCTT |
| *ACTB*  (human) | F: CATGTACGTTGCTATCCAGGC |
|  | R: CTCCTTAATGTCACGCACGAT |
